# A systematic review of spontaneous recovery in human fear conditioning

**DOI:** 10.3389/fnbeh.2026.1820847

**Published:** 2026-07-10

**Authors:** Joseph Anderson, Yi Wang, Dandan Li Clark, Ottmar V. Lipp, Madeline Jarvis, Luke J. Ney

**Affiliations:** School of Psychology and Counselling, Queensland University of Technology, Brisbane, QLD, Australia

**Keywords:** extinction recall, extinction retention, extinction retrieval, fear conditioning, spontaneous recovery

## Abstract

Spontaneous recovery is regarded as an experimental analogue of relapse following successful treatment of anxiety and traumatic disorders using exposure therapy. This systematic review examines the evidence for spontaneous recovery within human fear conditioning, highlighting its broad application in research and implications of findings for potential clinical implications. PubMed, Medline, and PsycINFO were searched from inception to 09/04/2026. In total, the search yielded 4,655 articles, of which 242 were included in the review. A meta-analysis was not conducted and the review was not preregistered. Evidence suggests differences in spontaneous recovery exist between clinical populations and healthy control groups, particularly for posttraumatic stress disorder, with mixed evidence observed in anxious versus healthy individuals. Our review reveals that paradigms involving a ~ 24-h delay between extinction and spontaneous recovery test (typically conducted across two consecutive days) are most frequently used and might produce the strongest effects. Importantly, we identify converging patterns between experimental findings and clinical outcomes between spontaneous recovery and the real-world efficacy of pharmacological interventions, providing preliminary support for the translational relevance of the paradigm. However, we note the slow uptake of non-pharmacological interventions in clinical trials despite promising experimental data and discuss findings from neurotransmitter and sleep studies that offer insights for optimizing exposure therapy. These findings suggest future research directions, including evaluating the correlation between experimental and clinical outcomes, exploring optimal conditions for the observation of spontaneous recovery, and understanding the effects of brain stimulation on fear conditioning.

## Introduction

Fear conditioning paradigms provide insights into the mechanisms underlying the acquisition and maintenance of human fears and can help us to understand trauma and anxiety-based behaviours (Beckers et al., 2023). These multi-phase paradigms commence with *acquisition* wherein a neutral stimulus is repeatedly paired with an unconditioned stimulus (US), such as an electrodermal shock, an air blast to the larynx, or aversive noise, to elicit a conditioned fear response (CR), thus creating a conditioned stimulus (CS+). Additional neutral stimuli are concurrently presented without the US (CS−) to provide a control or safety signal. Successful acquisition is defined by presence of CRs to the CS+ in the absence of the US that are not observed to the CS− (called differential conditioning). *Extinction* follows acquisition with repeated presentations of the CS+ and CS− without the US which results in the reduction and elimination of the CR (Myers and Davis, 2002). However, this does not imply that the fear association or ‘fear memory’ acquired during acquisition is resolved. Rather, the fear memory is thought to compete with the ‘extinction memory’ (Bouton, 2004; Lonsdorf et al., 2017). Fear conditioning is suggested to provide an experimental analogue for exposure therapy used to address phobias, anxiety, and stress related disorders such as posttraumatic stress disorder [PTSD; (Craske et al., 2014)], and has been suggested to be useful in research on fear relapse, which is frequent (Bisson et al., 2013; Levy et al., 2021) and remains an unresolved issue for the treatment of anxiety.

Return of conditioned fear can be observed by employing additional test phases post-extinction. *Reinstatement* tests present the CSs alone after the US was presented alone. *Reacquisition* is the re-presentation of CS+–US pairings. *Renewal* tests present the CSs in a context that differs from that of extinction (Wang et al., 2024). Finally, *recall* or ‘spontaneous recovery’ tests (see Box 1 for terminology) present the CSs again after the passage of some time, usually 24 h (Milad et al., 2005a). While the mechanisms of other relapse conditions are well explained (Bouton, 2002; Haaker et al., 2014; Wang et al., 2024), the mechanism underlying spontaneous recovery is less clear, though Bouton (2002) treated it as a special case of renewal due to the change in temporal context.

BOX 1 **Definitions of spontaneous recovery and extinction retention**

**Table:** 

Definitions	
Fear recall, return of fear, spontaneous recovery, fear retrieval	Fear extinction recall, extinction recall, extinction retention, extinction retrieval, extinction test
Fear returns simply due to the passage of time. Bouton (2002) describes as a special case of contextual renewal. Terms are often used interchangeably	The extinction memory—developed during the extinction phase (or exposure) is retained across time. It continues to compete with and inhibit the fear memory, regardless of the elapsed time

The measures used to determine spontaneous recovery usually are physiological, skin conductance response (SCR) and fear-potentiated startle (FPS), or subjective ratings (i.e., fear, arousal, US expectancy, CS valence). The opposing phenomenon of continued extinguished CRs is known as extinction retention (Lonsdorf et al., 2017). The extinction retention index (ERI) is a measure used to gauge the strength of an extinguished response and is calculated by comparing the difference between CRs towards the CS+ and CS− at the end of extinction (or in some studies acquisition) with the difference between the CRs to CS+ and CS− during the spontaneous recovery phase. In this way, the ERI can control for individual differences in extinction learning and how well the resulting extinction memory is retained at spontaneous recovery test (Milad et al., 2006; Milad et al., 2010). However, as ERIs can be calculated in a number of different ways, recent research has suggested that use of ERIs may increase researcher degrees of freedom, leading to less replicable results (Lonsdorf et al., 2019; Ney et al., 2018). A minimum of 24 h is typically employed between an extinction and spontaneous recovery test phase however, as noted by Lonsdorf et al. (2017) the effect of differing time gaps between extinction and recovery test is yet to be established. Moreover, the spontaneous recovery paradigm has been used in a very broad variety of ways and there is no common framework for how it should be conducted.

No study has yet collated and reviewed systematically how these variables influence spontaneous recovery, or how spontaneous recovery is used in research. Moreover, although the paradigm has now been used for two decades, there has been limited evaluation of its validity, coherence, or use in research, and no review has been conducted to date simply on the topic of the spontaneous recovery paradigm itself. The current review fills this gap by comprehensively describing how the spontaneous recovery paradigm has been used in fear conditioning. Our intention for the current article was to: (1) characterise how the paradigm is implemented across studies, (2) identify consistent and divergent findings across populations and methodologies, (3) evaluate the extent to which experimental results inform clinical understanding of fear relapse and treatment, and (4) identify methodological and conceptual gaps in the application of the paradigm.

The review is organized according to broader themes, which were identified after the search. First, we review brain imaging studies, which gives background information for later sections of the review. Next, we review the research on spontaneous recovery in clinical populations, including interventions that have been trialled using the spontaneous recovery paradigm. Next, we examine how spontaneous recovery paradigms have been used to explore new potential interventions (pharmacological, non-pharmacological, sleep, and brain stimulation) in healthy populations. Finally, we review other factors involved in spontaneous recovery, including underlying neurotransmitter systems, time delay between extinction learning and retention test, and other more general factors. The review concludes with a general discussion on the overarching findings relating to the themes identified above.

## Methods

### General procedure

**Figure 1:**
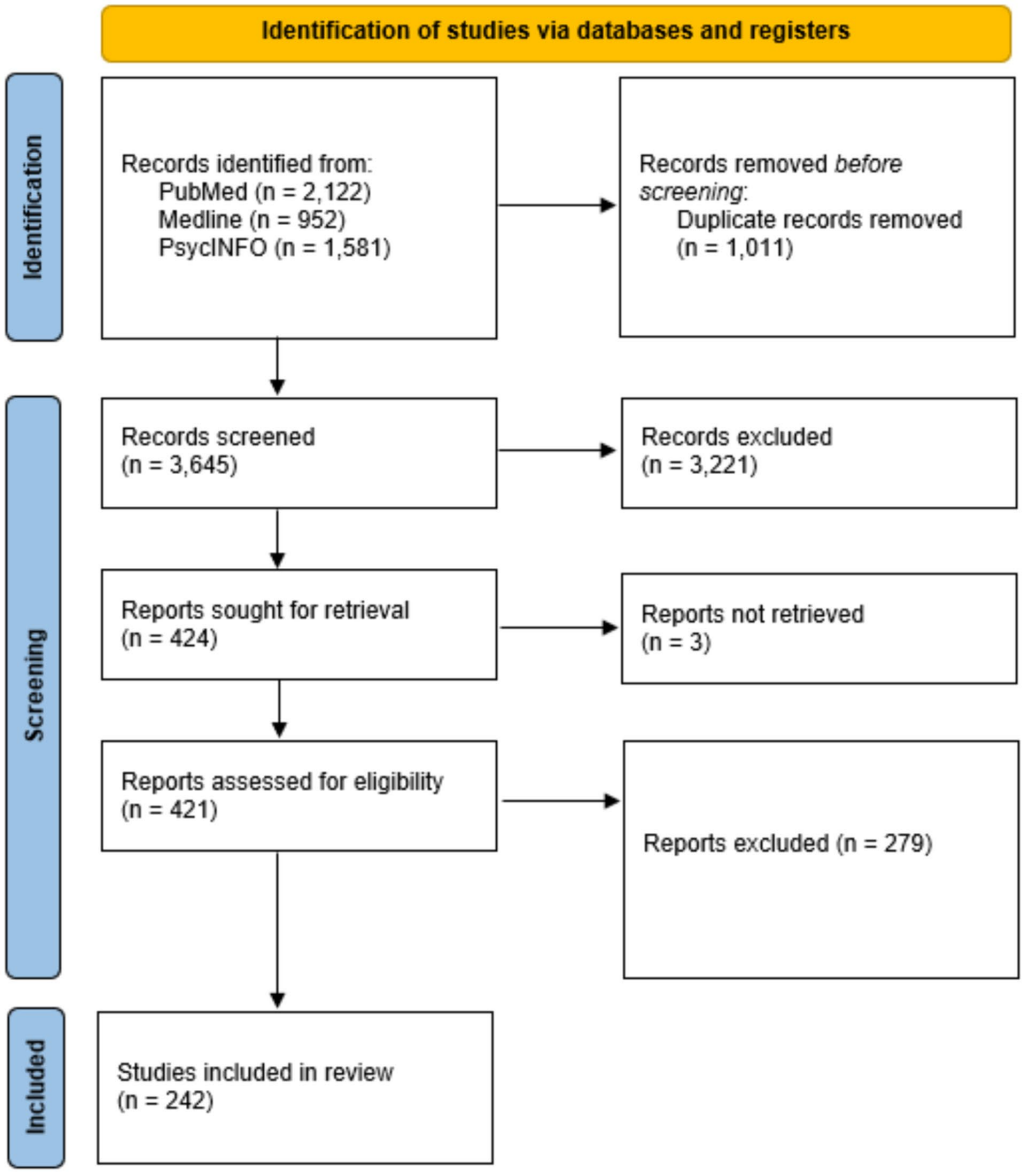
The screening process.

We conducted a systematic literature search according to PRISMA guidelines (Moher et al., 2015; Page et al., 2021). The search was performed ranging from each database’s earliest date until 17/04/2023. An updated search was conducted from this date until 09/04/2026. Articles that were found in the search were compiled and duplicates were removed in Endnote (The EndNote Team, 2013). Next, JA and YW independently screened articles based on their Title/Abstract and resolved any disagreements about study inclusions. JA and YW then independently screened the remaining articles based on their Full Texts and resolved any final disagreements about study inclusions. Conflicts around which articles were to be included were discussed and resolved by JA, YW, and LJN. Rayyan software (Ouzzani et al., 2016) was used to screen articles and identify conflicts between raters. In total, 4,655 articles were identified during our search, of which 1,011 duplicates were removed (Figure 1). 3,645 articles underwent Title/Abstract screening and 424 progressed to Full Text review. Following Full Text review, 242 articles were deemed to meet the criteria for inclusion in the systematic review. Cohen’s kappa (*κ*) values were calculated to determine agreement at each stage of the screening procedure: at the abstract screening stage agreement was κ ≈ 0.71 and at the full text screening stage agreement was κ ≈ 0.83. According to Landis and Koch (1977) this indicates substantial and almost perfect agreement.

Although this systematic review was not preregistered, several steps were taken to minimise potential selection and synthesis bias. These included the use of a comprehensive and predefined search strategy across multiple databases, clearly specified inclusion and exclusion criteria, and a structured screening process. Study selection and data extraction were conducted using standardised procedures, and decisions were guided by predefined criteria to reduce subjectivity. Furthermore, findings were synthesised systematically across studies, with attention to methodological differences and sources of heterogeneity to avoid selective reporting or overinterpretation of results.

### Search strategy

The systematic search of the literature utilised the PsychINFO, Medline (via Web of Science), and PubMed databases. The included search terms were ((fear conditioning OR threat conditioning OR fear acquisition OR threat acquisition OR fear extinction OR threat extinction OR extinction OR fear learning OR threat learning OR pavlovian conditioning OR conditioning, classical) AND (recall OR “return of fear” OR spontaneous recovery OR retention)). The comprehensiveness of the search strategy was evaluated by testing the search results against a list of known articles (20 in total). The search strategy was refined if all 20 articles were not identified by the search until we were satisfied that the final search strategy was able to identify a broad range of known fear conditioning articles assessing spontaneous recovery.

### Study eligibility

Articles in this review were required to meet the following inclusion criteria: (1) published in English, (2) journal articles, book chapters, or theses, (3) described the empirical use of a single cue or differential human fear conditioning paradigm, (4) the fear conditioning paradigm incorporated acquisition, extinction, and spontaneous recovery phases, where the spontaneous recovery phase involved a delay between extinction and spontaneous recovery, and spontaneous recovery was interpreted as being a measure of return of fear, and (5) the study reported the use of adult humans as participants. Many articles test both contextual renewal and spontaneous recovery. Articles for the current review were required to include a spontaneous recovery phase that did not involve contextual renewal.

Exclusion criteria for our systematic search included the following: (1) the article was a review or meta-analysis, (2) the article was not available in English, (3) the article only described secondary data, (4) the article used only child participants, (5) the article only used avoidance learning or evaluative conditioning, (6) the article only included delayed contextual renewal, and/or (7) the article did not include electrodermal responses, fear potentiated startle, functional magnetic resonance imaging (fMRI), or subjective ratings as outcome measures. Outcome measures were restricted to electrodermal activity, fear-potentiated startle, fMRI, and subjective ratings, as these represent the most established and validated indices of conditioned fear and its return in human paradigms (Lipp, 2006), thereby ensuring comparability across studies. However, we would note that the term “subjective ratings” refers to any type of rating that the participants made, whether it be evaluative (e.g., valence, arousal), fear ratings, US expectancy, or others.

### Data extraction and quality assessment

Data extraction was conducted using an extraction form to ensure consistency across studies. Key data items included study characteristics (author and date), number and type of participants, intervention components, intervention timing, number of trials during the spontaneous recovery phase, CS type, US type, time between extinction learning and spontaneous recovery, outcome measures, and main findings relevant to the review question. All details except for main findings are reported in tables and the main findings are summarised in the manuscript. Two reviewers independently extracted data (JA and LN). A methodological quality appraisal was conducted for all included studies using the Joanna Briggs Institute (JBI) critical appraisal tool (Supplementary material). Two reviewers independently assessed risk of bias, with discrepancies resolved through discussion.

### Systematic review structure

A meta-analysis was not conducted due to substantial conceptual, methodological, and statistical heterogeneity across the included studies, which precluded meaningful quantitative synthesis. The included studies varied markedly in their objectives, including but not limited to investigations of basic fear learning mechanisms, clinical translation, pharmacological modulation, neurobiological correlates, and individual difference factors. This diversity was accompanied by substantial variability in experimental design (e.g., conditioning procedures, timing of retention, reinforcement schedules), populations (clinical vs. non-clinical), outcome measures (behavioural, physiological, neural), and analytic approaches. As such, there was no common effect size or outcome metric that could be validly aggregated across studies.

Importantly, the heterogeneity observed was not simply statistical but conceptual, with many studies addressing fundamentally different research questions using the extinction retention paradigm as a tool rather than evaluating a shared intervention or outcome. Under these conditions, conducting a meta-analysis—including subgroup or multilevel approaches—would risk producing misleading or uninterpretable summary estimates. Finally, nearly all studies assessed a unique manipulation of some kind, meaning that even a basic meta-analysis of un-manipulated spontaneous recovery would not be possible.

Throughout the article, included studies are summarized in tables. In these tables, use of the ERI and other corrections to differences in extinction are flagged. While we did not extract the exact formulae used to calculate ERIs and other corrections, we denote in the tables what type of correction was used, whether it was ERI, a correction based on extinction learning, or a correction based on acquisition learning. The latter two types of corrections were denoted separately from ERIs where they did not generate a single value (e.g., percentage of fear return or extinction retention), but were instead included as part of the standard analysis, e.g., repeated measure ANOVA. Where available, effect sizes are reported.

## Results and discussion

### Brain imaging

Enhanced activity of the ventromedial prefrontal cortex (vmPFC) and other higher order brain areas are hypothesised to play a major role in inhibiting threat responses from the amygdala and other mid-brain areas involved in fear, thereby contributing to the development of extinction memories during extinction learning (Bouton et al., 2020; Phelps et al., 2004; Quirk and Mueller, 2008). Using fMRI, Åhs et al. (2015) found that vmPFC activity was higher in response to the CS− compared to CS+ during spontaneous recovery, and that the left as well as right hippocampus displayed significantly lower reactivity in response to the CS+ (Table 1). Similarly, higher vmPFC activity during extinction retention (as well as lower dorsomedial PFC and anterior cingulate cortex [ACC] activity) was associated with higher cognitive reappraisal (Hermann et al., 2014). Hermann et al. (2016), Hermann et al. (2020), and Sperl et al. (2019) reported reductions in hippocampus and amygdala activity as well as higher activation in the left insula during extinction retention, and higher connectivity between hippocampus and amygdala was associated with increased spontaneous recovery (Hermans et al., 2017). Gold et al. (2020) and Widegren et al. (2025) reported that effects of vmPFC-amygdala connectivity during extinction retention were moderated by age. Lebron-Milad et al. (2012) reported sex differences in brain activation during extinction retention, with male participants showing higher activation of the rostral anterior cingulate cortex (rACC) and vmPFC, while females had greater insula activation.

**Table 1:** Use of spontaneous recovery paradigms with functional magnetic resonance imaging.

Study	Sample size	CS	US	fMRI timing	Areas implicated during recall	Trials during recall	Extinction—recall delay
Åhs et al. (2015)	43	Spiders, snakes	Shock	Acquisition, extinction, recall, renewal	vmPFC, amygdala, hippocampus	16	24 h
Batsikadze et al. (2022)	31	Lamp colours	Shock	Acquisition, extinction, recall, renewal	Cerebellum	8	24 h
Batsikadze et al. (2024)	41	Lamp colours	Shock	Acquisition, extinction, recall	Cerebellum	8	24 h
Bauer et al. (2025)	53	Animals, tools, food (categories)	Shock	Acquisition, extinction, recall	vmPFC, dACC	120	24 h, 1 month
Cushing et al. (2024)	223	Lamp colours	Shock	Acquisition, extinction, recall	dACC, mPFC, inferior frontal gyrus	8	48 h
de Voogd et al. (2018)	24	Coloured squares	Shock	Acquisition, extinction, recall, reinstatement	Insula, dACC	6	24 h
Dunsmoor et al. (2019)	46	Angry faces	Shock	Acquisition, extinction, recall	vmPFC, amygdala, insular, sgACC, posterior parietal cortex, thalamus, striatum, cingulate cortex	12	24 h
Ebrahimi et al. (2020)	37	Neutral faces	Scream	Acquisition, recall	Amygdala, hippocampus, dACC, insula	24	24 h
							
Feng et al. (2016)	40	Coloured squares	Pictures	Acquisition, extinction, reactivation, recall	vmPFC, amygdala	60	24 h
Garfinkel et al. (2014)	28	Lamp colours	Shock	Acquisition, extinction, recall, renewal	vmPFC, mPFC, amygdala, insula, thalamus, occipital lobe, and parietal cortex	8	24 h
Glenn et al. (2021)	36	Blue and yellow bells, neutral faces	Scream or alarm	Recall	vmPFC, anterior insular cortex, amygdala, dmPFC, dACC	24	3 weeks
Gold et al. (2020)	200	Neutral faces	Scream	Recall	vmPFC, amygdala, inferior temporal cortex	24	3 weeks
Gong et al. (2025)	96	Coloured squares	Shock	Acquisition, extinction, recall	Hippocampus, thalamus, and precuneus	15	24 h, 2 weeks
Haaker et al. (2015)	41	Shapes	Shock	Recall, reinstatement	dmPFC, hippocampus, amygdala	6	1 week
Hagedorn et al. (2022)	60	Shapes	Shock	Acquisition, extinction, recall	Parahippocampal gyrus, amygdala, hippocampus	4	24 h
Helpman et al. (2016)	58	Lamp colours	Shock	Recall	vmPFC, rACC, sgACC, hippocampus, parahippocampus	8	24 h
Hermann et al. (2014)	41	Neutral faces	Film clips	Acquisition, extinction, recall	vmPFC, anterior cingulate, dmPFC	12	24 h
Hermann et al. (2016)	46	Coloured lamps	Shock	Acquisition, extinction, recall, renewal	vmPFC, hippocampus, insula, amygdala	8	24 h
Hermann et al. (2017)	45	Coloured lamps	Shock	Diffusion tensor imaging during recall and renewal	Right uncinate fasciculus	8	24 h
Hermann et al. (2020)	49	Coloured lamps	Shock	Acquisition, extinction, recall, renewal	Hippocampus	8	6–8 days
Hermans et al. (2017)	24	Neutral faces	Shock	Acquisition, extinction, recall	Amygdala, hippocampus	16	24 h
Jacoby et al. (2025)	17	Coloured lamps	Shock	Acquisition, extinction, recall	Left Supramarginal Gyrus	8	24 h
Krause-Utz et al. (2016)	43	Coloured shapes	Shock	Acquisition, extinction, recall, re-acquisition	Lingual gyrus, cuneus, frontal gyrus, parahippocampal gyrus, temporal gyrus, cerebellum	18	3 days
Kroes et al. (2016)	46	Lamp colours	Shock	Extinction, recall	Midbrain, hippocampus, amygdala, and dmPFC	12	24 h
Lange et al. (2019)	48	Shapes	Shock	Acquisition, generalization, extinction, recall	Precuneus cortex	12	24 h
Lange et al. (2020)	45	Shapes	Shock	Extinction, recall	None	12	24 h
Lebron-Milad et al. (2012)	24	Coloured lamps	Shock	Acquisition, extinction, recall	rACC, insula	Not reported	24 h
Linnman et al. (2012)	18	Coloured lamps	Shock	Acquisition, extinction, recall	vmPFC, amygdala, dACC	8	24 h
Lonsdorf et al. (2014)	39	Shapes	Shock	Recall, reinstatement	vmPFC	6	6 days
Marin et al. (2016)	65	Lamp colours	Shock	Recall	vmPFC, amygdala, dACC, hippocampus,	8	24 h
Marin et al. (2017)	82	Lamp colours	Shock	Acquisition, recall	vmPFC, rACC, insular cortex	8	24 h
Merz et al. (2018)	40	Lamp colours	Shock	Extinction, recall, renewal	vmPFC, amygdala, insula	8	24 h
Milad et al. (2005a), Milad et al. (2005b)	14	Coloured lamps	Shock	Recall, renewal	Medial orbitofrontal cortex (vmPFC)	5	24 h
Milad et al. (2007)	14	Coloured lamps	Shock	Acquisition, extinction, recall	vmPFC, hippocampus, amygdala	8	24 h
Milad et al. (2009)	31	Lamp colours	Shock	Acquisition, extinction, recall	vmPFC, hippocampus, dACC	8	24 h
Neudert et al. (2023)	72	Lamp colours	Shock	Acquisition, extinction, recall, renewal	none	8	24 h
Pejic et al. (2013)	41	Neutral faces	Critical film clips	Acquisition, extinction, recall	vmPFC (marginal)	12	24 h
Peng et al. (2023)	265	Lamp colours	Shock	Acquisition, extinction, recall	sgACC	8	1–7 days
Rabinak et al. (2014)	28	Coloured shapes	Loud noise	Extinction, recall	vmPFC, hippocampus	20	24 h
Rabinak et al. (2018)	75	Coloured shapes	Loud noise	Resting, recall	vmPFC, amygdala, hippocampus	20	24 h
Rougemont-Bücking et al. (2011)	34	Lamp colours	Shock	Acquisition, extinction, recall	dACC, vmPFC, frontal cortex, middle frontal gyrus	8	24 h
Rousseau et al. (2020)	37	Shapes	Shock	Extinction, recall	Precuneus, medial prefrontal gyrus	48	24 h
Schwarzmeier et al. (2019)	20	Neutral faces	Scream	Extinction, recall	middle frontal gyrus, inferior frontal gyrus, inferior frontal operculum, insula	8	24 h
Seo et al. (2018)	46	Lamp colours	Shock	Acquisition, extinction, recall	vmPFC, dACC, insula	8	24 h
Seo et al. (2022)	126	Lamp colours	Shock	Acquisition, extinction, recall	rACC, hippocampus, insula, supplementary motor area	8	24 h
Seo et al. (2026)	30	Lamp colours	Shock	Acquisition, extinction, recall	vmPFC, dlPFC, DMN, amgydala, OFC, SMG, hippocampus	8	24 h
Shvil et al. (2014)	56	Lamp colours	Shock	Acquisition, extinction, recall	rACC	8	24 h
Spencer et al. (2017)	40	Lamp colours	Shock	Extinction, recall	vmPFC, hippocampus	8	24 h
Sperl et al. (2019)	18	Neutral faces	Shock	Acquisition, extinction, recall	Amygdala	40	24 h
Spohrs et al. (2022)	55	Shapes	Thermal	Acquisition, extinction, recall	dACC, insular, temporal gyrus, postcentral gyrus, caudate nucleus	20	24 h
Spoormaker et al. (2010)	16	Shapes	Shock	Extinction, recall	vmPFC, bilateral lingual gyrus	15	4 h
Spoormaker et al. (2012)	18	Shapes	Shock	Extinction, recall	temporal gyrus, amygdala, occipital gyrus, putamen	15	24 h
Steiger et al. (2015)	37	Coloured square	Shock	Acquisition, extinction, recall	none	9	24 h
Sun (2019)	190	Lamp colours	Shock	Recall	none	8	24 h
Wen et al. (2021)	90	Lamp colours	Shock	Extinction, recall	vmPFC, hippocampus	8	24 h
Wen et al. (2022)	435	Lamp colours	Shock	Acquisition, extinction, recall	Amygdala, inferior parietal gyrus	8	24 h
Wen et al. (2026)	59	Lamp colours	Shock	Acquisition, avoidance, extinction, recall	None reported	8	24 h
Widegren et al. (2025)	120	Neutral faces	Scream	Recall	ACC, insular cortex, amygdala	10	24 h
Zabik et al. (2021)	59	Coloured bag images	Snake (virtual reality)	Acquisition, extinction, recall	dACC	20	24 h
Zabik et al. (2023)	77	Coloured lights	Loud noise	Acquisition, extinction, recall, renewal	Hippocampus	20	24 h
Zhou et al. (2025)	64	Coloured shapes	Shock	Extinction, recall	dmPFC	16	24 h

Although most studies are broadly in agreement about how extinction retention is operationalised on a neural level, the details of the relationship are complicated. For example, vmPFC activity during spontaneous recovery was negatively associated with resting amygdala activity (Linnman et al., 2012), but Rabinak et al. (2018) found no correlation between spontaneous recovery, vmPFC activity, and amygdala-hippocampal functional connectivity measured during resting state. In contrast to Åhs et al. (2015), some studies have also reported higher vmPFC activation to CS+ in participants with lower physiological return of fear (Lonsdorf et al., 2014; Milad et al., 2005b; Milad et al., 2007); and no CS+/CS− neural differences were identified during extinction retention in at least two studies (Krause-Utz et al., 2016; Neudert et al., 2023).

Some studies have also argued that other brain areas are critical to extinction retention. Compared to CS−, higher posterolateral cerebellum activation in response to the CS+ was observed during contextual renewal (but not extinction retention), suggesting that fear memories may also remain in the cerebellum during extinction (Batsikadze et al., 2022), and Cushing et al. (2024) argued that data from 223 participants suggested broader networks—rather than isolated brain regions—are involved in extinction retention (Jacoby et al., 2025). Using diffusion tensor imaging, Hermann et al. (2017) found that high spontaneous recovery was associated with lower fractional anisotropy—meaning smaller size and lower number of myelinated axons and reduced structural integrity—in the right uncinate fasciculus.

Different types of interventions and paradigms also suggest differential networks and brain regions are involved in extinction retention. Activation of the precuneus and surrounding networks was observed during recall when bilateral auditory stimulation—analogous to eye-movement desensitisation and reprocessing by presentation of auditory tones alternatively to the left and right ears at a rate of one hertz—was conducted during extinction (Rousseau et al., 2020). Finally, a novel virtual reality paradigm found differences in hippocampal activation during recall—potentially signalling differences in contextual information processing—compared to standard fear conditioning (Zabik et al., 2023).

**Table:** 

Summary—brain imaging
vmPFC activity has been centrally implicated in facilitating extinction retention. This likely reflects top-down inhibition of the fear response, which is associated with amygdala activity
Hippocampus, anterior cingulate cortex, insula, and other brain regions are also implicated in extinction retention and/or spontaneous recovery
However, it is worthwhile noting that, for the most part, fMRI studies have focused on a limited number of brain regions, not all studies replicate, and sample sizes are normally modest

### Clinical populations

Higher levels of spontaneous recovery during fear conditioning paradigms have often been linked to the presence of different mental health disorders (Table 2).

**Table 2:** Use of spontaneous recovery paradigms with different clinical groups.

Study	Sample size	CS	US	Manipulation	Manipulation timing	Trials during recall	Extinction—recall delay	Outcome measures
Acheson et al. (2015)	51	Yellow and blue circles	Air blast	Bipolar versus healthy control	n/a	8	24 h	MRI, FPS, expectancy*
Barnes-Horowitz et al. (2023)	36	Neutral faces	Scream	Positive and Negative Affect Treatment in anxiety and depression	Treatment took place after recall	4	12 days	SCR, expectancy
Cano et al. (2021)	36	Lamp colours	Shock	OCD versus healthy control, CBT treatment	Treatment took place after recall	8	24 h	fMRI, SCR
Cisler et al. (2020)	91	Shapes	Shock	L-DOPA versus placebo. All participants had PTSD	L-DOPA or placebo after extinction, Context was manipulated during recall	6	24 h	SCR, fMRI^
Crombie et al. (2021)	35	Shapes	Shock	Exercise versus control. All participants had PTSD	Exercise or control completed after extinction	10	24 h	SCR, expectancy
de Vos et al. (2025)	43	Circles	Shock	Exposure therapy versus progressive relaxation for spider phobia	Pre-exposure conditioning correlated with long-term therapy outcome	12	24 h	fMRI, fear, expectancy
Fricke et al. (2024)	108	Lamp colours	Shock	SAD versus healthy control	n/a	8	24 h	SCR, fMRI
Fyer et al. (2020)	166	Lamp colours	Shock	OCD, SAD, anorexia versus healthy control	n/a	5	24 h	SCR
Garfinkel et al. (2014)	28	Lamp colours	Shock	Veterans with PTSD versus combat control	n/a	8	24 h	SCR, fMRI
Giménez et al. (2020)	30	Lamp colours	Shock	OCD versus healthy control, CBT treatment	Treatment took place after recall	8	24 h	SCR, fMRI*
Glotzbach-Schoon et al. (2015)	42	VR context –office rooms	Shock	Reinstatement versus non-reinstatement, Anxiety measured	Reinstatement (or not prior to recall)	4	24 h	SCR, FPS, anxiety, expectancy*
Gold et al. (2020)	200	Neutral faces	Scream	Anxiety versus healthy control, generalization	Generalization during recall	24	3 weeks	SCR, fMRI, threat appraisal, explicit memory
Helpman et al. (2016)	58	Lamp colours	Shock	PTSD versus trauma control, prolonged exposure	Recall completed before and after prolonged exposure	8	24 h	SCR, fMRI
Hofmann et al. (2019)	81	Lamp colours	Shock	DCS versus placebo in SAD	DCS or placebo prior to extinction	8	24 h	SCR, expectancy
Holt et al. (2012)	37	Lamp colours	Shock	Schizophrenia versus healthy control	n/a	8	24 h	SCR, fMRI*
Holt et al. (2009)	46	Lamp colours	Shock	Schizophrenia versus healthy control	n/a	5	24 h	SCR*
Krause-Utz et al. (2016)	43	Coloured shapes	Shock	Bipolar versus healthy control	n/a	18	3 days	SCR, fMRI, valence, arousal
Lange et al. (2019)	48	Shapes	Shock	Spider phobia versus healthy controls. Generalization	Generalization after acquisition and during recall	12	24 h	fMRI, fear ratings, expectancy
Lange et al. (2020)	45	Shapes	Shock	Exposure therapy versus progressive muscle relaxation. Spider phobia. Generalization	Treatment 4 weeks after recall. Generalization during recall	12	24 h	fMRI, fear ratings, expectancy
Maples-Keller et al. (2022a), Maples-Keller et al. (2022b)	123	Coloured shapes	Air blast	PTSD versus trauma control. Prolonged exposure	Recall took place after treatment	4	24 h	FPS
Marin et al. (2020)	114	Lamp colours	Shock	Anxiety disorders versus healthy control	n/a	8	24 h	SCR, fMRI
Marin et al. (2016)	65	Lamp colours	Shock	PTSD versus trauma control and non-trauma control	n/a	8	24 h	SCR, fMRI, PET*
McLaughlin et al. (2015)	55	Lamp colours	Shock	OCD versus healthy control	n/a	5	24 h	SCR*
Milad et al. (2013)	42	Lamp colours	Shock	OCD versus healthy control	n/a	8	24 h	SCR, fMRI
Milad et al. (2008)	28	Lamp colours	Shock	Monozygotic twins—PTSD versus trauma versus non-trauma control	n/a	5	24 h	SCR*
Milad et al. (2009)	31	Lamp colours	Shock	PTSD versus trauma control	n/a	8	24 h	SCR, fMRI*
Norrholm et al. (2025)	51	Shapes	Air blast	PTSD versus controls in female military veterans	n/a	4	24 h	FPS, expectancy^
Pöhlchen et al. (2020)	226	Shapes	Shock and air blast	Fear related disorders versus control	n/a	8	24 h	SCR, FPS, pupil, expectancy
Pöhlchen et al. (2021)	24	Shapes	Shock	OCD versus healthy control	n/a	5	24 h	SCR, FPS, pupil, expectancy
Pejic et al. (2013)	41	Neutral faces	Critical film clips	Healthy, completed SAD measure	n/a	12	24 h	SCR, fMRI, valence, arousal
Peng et al. (2023)	265	Lamp colours	Shock	Anxiety, depression versus healthy control	n/a	8	1–7 days	SCR, fMRI
Rosenbaum et al. (2015)	222	Coloured lamps	Shock	OCD, PTSD, schizophrenia versus control. Demographics assessed	n/a	8	24 h	SCR
Rougemont-Bücking et al. (2011)	34	Lamp colours	Shock	PTSD versus trauma control	n/a	8	24 h	fMRI
Schwarzmeier et al. (2019)	20	Neutral faces	Scream	Panic disorder versus healthy control	n/a	8	24 h	SCR, fMRI, valence, arousal
Shvil et al. (2014)	56	Coloured lamps	Shock	PTSD versus trauma control	n/a	8	24 h	SCR, fMRI*
Seo et al. (2018)	46	Lamp colours	Shock	Insomnia versus good sleepers	PSG measured prior to conditioning	8	24 h	SCR, fMRI
Seo et al. (2022)	126	Lamp colours	Shock	PTSD versus trauma controls	PSG measured prior to conditioning and after extinction	8	24 h	SCR, fMRI, expectancy
Spencer et al. (2017)	40	Lamp colours	Shock	ADHD versus healthy control	n/a	8	24 h	SCR, fMRI*
Steiger et al. (2015)	37	Coloured square	Shock	PTSD versus trauma and non-trauma controls	n/a	9	24 h	fMRI, expectancy, arousal, valence
Steinman et al. (2022)	67	Angry faces	Shock	OCD, GAD versus phobia	n/a	10	24 h	SCR^
Straus et al. (2018)	13	Coloured circles	Air blast	Veterans with PTSD, PSG measured	PSG measured before and after extinction	8	24 h	FPS*
Van't Wout et al. (2017)	28	Coloured lamps	Shock	tDCS with no control. All participants had PTSD	During extinction or after extinction	8	24 h	SCR
Vanuk et al. (2022)	82	Coloured cars	Shock	PTSD, blue light treatment	Blue light 6 weeks prior to recall	16	6 weeks	SCR, fMRI, heart rate*
Wicking et al. (2016)	54	Shapes	Shock	PTSD versus trauma and non-trauma controls	Context change to recall	10	7 days	SCR, expectancy

*Use of extinction retention index, ^use of correction to extinction learning. ADHD, attention-deficit, hyperactivity disorder; CBT, cognitive behavioural therapy; DCS, D-cycloserine; fMRI, functional magnetic resonance imaging; FPS, fear potentiated startle; GAD, general anxiety disorder; h, hours; L-DOPA, levodopa; OCD, obsessive compulsive disorder; PET, positron emission tomography; PSG, polysomnography; PTSD, posttraumatic stress disorder; SAD, social anxiety disorder; SCR, skin conductance response; tDCS, transcranial direct current stimulation.

#### Post-traumatic stress disorder

*Psychophysiology and ratings*. Six studies have compared populations of combat-exposed war veterans with diagnosed PTSD and combat exposed veterans without PTSD. Milad et al. (2008) noted that veterans with PTSD were the only group to exhibit fear recall compared to their non-military monozygotic twin, combat-exposed veterans without PTSD, and the non-military twins of the non-PTSD veterans. Analysis of ERI indicated that veterans with PTSD trended (*p* = 0.06, not significant) toward weaker ERI than veterans without PTSD and had significantly weaker ERI than their non-trauma-exposed twin (Milad et al., 2008). Non-twin studies provide stronger evidence for difficulties for PTSD veterans in retaining extinction memories using SCR (Garfinkel et al., 2014; Milad et al., 2009; Vanuk et al., 2022), though Straus et al. (2018) found successful extinction retention of fear potentiated startle (FPS) in veterans with PTSD and Norrholm et al. (2025) found no differences between females with military sexual trauma with and without PTSD during spontaneous recovery.

An additional fourteen studies in non-veteran PTSD populations have produced mostly positive results, although these studies differed in population type, control groups, and study design (Bauer et al., 2025; Cisler et al., 2020; Crombie et al., 2021; Marin et al., 2016; Rougemont-Bücking et al., 2011; Seo et al., 2022; Steiger et al., 2015; Wicking et al., 2016). However, Pöhlchen et al. (2020) was not able to find any differences between spontaneous recovery in PTSD participants or participants with fear-related disorders compared to healthy controls (*η_p_^2^* = 0.04). Schenker et al. (2022) also observed a uniform SCR increase during extinction retention, however noted that for women with high PTSD symptom severity, better quality subjectively-reported sleep correlated with stronger extinction retention, a result that was supported by subsequent meta-analysis (Schenker et al., 2021).

Brain imaging. Supporting the role of the vmPFC in extinction retention, Garfinkel et al. (2014) found that non-PTSD combat control participants had significantly stronger vmPFC activation during extinction test when compared to PTSD patients (Garfinkel et al., 2014). Studies have found that anxious, distressed, and PTSD participants show reduced vmPFC and greater subgenual, rostral anterior cingulate cortex (rACC), and dorsal anterior cingulate cortex (dACC) activation during extinction retention (Bauer et al., 2025; Cushing et al., 2024; Marin et al., 2016; Milad et al., 2009; Pejic et al., 2013; Peng et al., 2023; Rougemont-Bücking et al., 2011; Seo et al., 2022; Spencer et al., 2017), signifying less top-down control of fear memories in these populations (reported effect size η_p_^2^ = 0.15). Male patients with PTSD had higher rACC activation during recall compared to female patients with PTSD (Shvil et al., 2014), though these findings were not replicated by Wen et al. (2022) who only found increased amygdala activity to CSs in females when compared to males during spontaneous recovery.

*Interventions*. Combining cognitive treatments with fear conditioning, Helpman et al. (2016) and Maples-Keller et al. (2022b) assessed the long-term effect of prolonged exposure treatment in PTSD clients. Helpman et al.’s analysis of SCR responses pre and post 10-week treatment indicated a non-significant improvement in extinction retention for the PTSD group, but an increase in functional connectivity between vmPFC, rACC, and subgenual ACC, suggesting improved top-down control of these areas with exposure therapy treatment. On the other hand, Maples-Keller et al. identified low versus high responders to prolonged exposure treatment and found that extinction retention (using FPS) was evident in the high, but not low, treatment response group (*η^2^* = 0.12). Cisler et al. (2020), Van't Wout et al. (2017), and Crombie et al. (2021) administered L-DOPA (versus placebo), tDCS (versus sham), and moderate intensity exercise (versus control) prior to extinction, respectively, to participants with PTSD. None of these studies, however, reported benefits of these interventions on spontaneous recovery. By contrast, in a group of participants with PTSD, Vanuk et al. (2022) reported that 6-weeks of blue (compared to amber) light therapy prior to extinction resulted in significantly improved extinction retention (*η_p_^2^* = 0.05).

#### Anxiety and obsessive-compulsive disorder

*Psychophysiology and ratings*. Two studies have found no effect of anxiety levels in non-clinical populations on fear extinction retention. Glotzbach-Schoon et al. (2015) measured non-clinical anxiety levels during a three-day contextual fear conditioning task, but found that anxiety levels did not correlate with any outcomes measure. Similarly, Pejic et al. (2013) found that social anxiety levels to have no impact on spontaneous recovery of valence, arousal, and fear ratings. Results from pathologically anxious populations seem to support these findings. Successful extinction retention in participants with panic disorder (Schwarzmeier et al., 2019) and similar extinction retention (measured by SCRs) in healthy controls and participants with social anxiety disorder (Fyer et al., 2020) further refute the hypothesis that anxious participants ubiquitously show higher spontaneous recovery. Likewise, significantly higher SCRs to CS− than CS+ indicated extinction retention for anxious, depressed, and co-morbid anxious and depressed participants, though a typical fear recall response (CS+ larger than CS−) returned during a 30-month follow-up test (Peng et al., 2023).

Six studies of obsessive-compulsive disorder (OCD) have compared OCD to controls during spontaneous recovery, but consistent results have not been produced. Marginally significant results in Fyer et al. (2020) and Milad et al. (2013) showed trends towards differential spontaneous recovery of fear in OCD when contrasted with healthy control participants, while McLaughlin et al. (2015) observed equivalent spontaneous recovery of fear in both control and OCD groups. For the controls this was observed on the first trial, while for OCD, this was observed on the second trial which yielded stronger differential response magnitudes. Follow-up ERI was suggestive of comparatively weaker extinction retention for OCD participants, *d* = 0.89 (McLaughlin et al., 2015). Pöhlchen et al. (2021), Cano et al. (2021), and Giménez et al. (2020) also found no difference between OCD and control participants during spontaneous recovery.

*Brain imaging*. Some studies suggest that anxiety may moderately affect brain activity despite no changes to behaviour or physiology during spontaneous recovery in anxiety. Across social anxiety disorder, generalised anxiety, panic disorder, and social phobia, Marin et al. (2017) did not find differences in physiological spontaneous recovery as a function of diagnosis, though this study found higher activity in the amygdala of anxious patients compared to healthy controls during spontaneous recovery, with healthy participants showing strong vmPFC-subgenual ACC connectivity (Marin et al., 2017). Likewise, some neural differences were found in Lange et al. (2019), who reported no differences between CS+ and CS− during extinction retention in the precuneus of healthy controls, though reduction of activation towards the CS+ was observed in spider phobic participants during phobia-irrelevant conditioning. Finally, higher activation in the inferior frontal gyrus and operculum, as well as insula, was reported during extinction retention in patients with panic disorder when compared to healthy controls (Schwarzmeier et al., 2019). Moreover, Gold et al. (2020) found reduced vmPFC activity in anxious young participants compared to controls during spontaneous recovery (*d* = 0.64), and Fricke et al. (2024) found that differential amygdala activation in socially anxious participants compared to controls.

*Interventions*. Mixed findings also have been reported for studies examining the relationship between spontaneous recovery and interventions for anxiety. Neither fear ratings, fMRI signal, or US expectancy measured during spontaneous recovery predicted short- or long-term therapy outcome in spider phobics undergoing exposure therapy (de Vos et al., 2025; Lange et al., 2020). Another study found that US expectancy, but not SCR or FPS, during extinction retention exhibited a positive relationship with exposure therapy outcome for spider phobia (Forcadell et al., 2017b). A test of the effect of novelty-facilitated extinction on extinction retention in an anxious participant group was inconclusive, as within-session extinction was not achieved (Steinman et al., 2022). Efficacy studies of CBT-related interventions, however, have suggested that higher extinction retention for OCD participants correlates with successful therapy outcome (Cano et al., 2021; Giménez et al., 2020), though, relative to placebo, d-cycloserine administration did not reduce fear recall to either an extinguished or unextinguished CS+s in a cohort of socially anxious participants (Hofmann et al., 2019).

#### Other populations

Spontaneous recovery has been explored in other psychiatric conditions. Successful extinction retention of SCRs in a standard fear conditioning paradigm has been noted for those living with attention-deficit/hyperactivity disorder (Spencer et al., 2017). Conversely, consistently high spontaneous recovery was observed in schizophrenic individuals (Holt et al., 2012; Holt et al., 2009). Successful extinction retention and significantly stronger ERI for participants with bipolar disorder compared to healthy controls led Acheson et al. (2015) to suggest that mood stabilisers and other psychotropic medications may play a role in altering memory traces. Krause-Utz et al. (2016) that an unmedicated bipolar sample showed a non-significant trend (*p* = 0.089) towards larger spontaneous recovery of fear in SCR and no group differences were evident in arousal or valence ratings. Interestingly, Seo et al. (2018) found evidence for higher vmPFC, dACC, and insula activation during extinction retention in patients with insomnia compared to good sleepers, which contradicts evidence found in healthy populations compared to other clinical cohorts such as PTSD.

Finally, a recent study by Barnes-Horowitz et al. (2023) reported that Positive Affective Treatment for depression was associated with better treatment outcomes in participants who had higher extinction retention, whereas Negative Affective Treatment was associated with better treatment outcomes in participants who had higher spontaneous recovery. This study highlights how spontaneous recovery may discriminate categories of patient phenotypes.

In summary, spontaneous recovery has mostly been shown to differ between participants with and without PTSD, however, this relationship does not appear to be consistent in other anxiety disorders. Emerging research suggests that part of this issue may be due to overlap of phenotypes within anxious participant groups. Comparisons directly between different clinical groups are highly encouraged for future research.

**Table:** 

Summary—clinical populations
Higher spontaneous recovery in PTSD compared to trauma- and non trauma-exposed controls
Minimal evidence for differences in spontaneous recovery between controls and participants with other anxiety disorders, such as OCD and general anxiety
Specific participant anxiety phenotypes may moderate the relationship between spontaneous recovery and mental disorders
More evidence is needed to demonstrate the utility of spontaneous recovery as a tool to discriminate participants with mental disorders from controls

### Pharmaceutical interventions

Pharmaceutical studies of fear conditioning have explored the impact of both synthetic drugs and endogenous substances on extinction retention in healthy participants (Table 3). This use of the spontaneous recovery paradigm is an attempt to understand what types of medications may be efficacious in reducing return of fear. Across two experiments, Gerlicher et al. (2019) found that administration of Levodopa (L-DOPA) resulted in stronger extinction retention, though only in participants with low SCRs at the end of extinction. This was not replicated in Andres et al. (2024) or Doubliez et al. (2025). Haaker et al. (2015) also assessed L-DOPA’s effect, though in this study individuals with lingering CS+/CS− discrimination at end of extinction maintained this differential responding at spontaneous recovery regardless of drug condition (Haaker et al., 2013a; Haaker et al., 2015).

**Table 3:** Use of spontaneous recovery paradigms with pharmacological interventions.

Study	Sample size	CS	US	Manipulation	Manipulation timing	Trials during recall	Extinction—recall delay	Outcome measures
Acheson et al. (2013)	44	Blue and yellow circles	Shock	Oxytocin versus placebo	After acquisition and 1.25 h prior to extinction	8	24 h	FPS, expectancy, anxiety*
Andres et al. (2024)	55	Shapes	Shock	L-DOPA versus placebo	L-DOPA or placebo after extinction	10	24 h	SCR, expectancy, fMRI
Cisler et al. (2020)	91	Shapes	Shock	L-DOPA versus placebo	L-DOPA or placebo after extinction, Context was manipulated during recall	6	24 h	SCR, fMRI^
Culver et al. (2014)	70	Coloured shapes	Scream	Caffeine versus placebo	Caffeine or placebo prior to extinction	4	24 h	SCR, expectancy, valence^
Das et al. (2013)	48	Red and yellow boxes	Shock	Cannabidiol versus placebo	Cannabidiol before or after extinction	16	24 h	SCR, expectancy
Doubliez et al. (2025)	146	Shapes	Shock	L-DOPA, bromocriptine, tiapride, haloperidol versus placebo	60–120 min before extinction	8	24 h	SCR, pupil size
Ebrahimi et al. (2020)	37	Neutral faces	Scream	DCS versus placebo	DCS or placebo 1 h before extinction	24	24 h	SCR, fMRI, valence, arousal^
Gerlicher et al. (2019)	79, 32	Shapes	Shock	L-DOPA versus placebo, long versus short extinction	L-DOPA or placebo after extinction	8	24 h	SCR, FPS, expectancy, fear ratings
Graham and Milad (2013)	76	Coloured lamps	Shock	Hormonal contraceptive versus naturally cycling	n/a	8	24 h	SCR*
Guastella et al. (2007)	97, 44	Coloured blocks	Shock	DCS versus placebo	DCS or placebo before extinction	1	24 h	SCR, expectancy
Haaker et al. (2013a); Haaker et al. (2013b)	39	Shapes	Shock	L-DOPA versus placebo	L-DOPA or placebo after extinction, Context was manipulated during recall	24	24 h	SCR, fMRI, expectancy, fear ratings
Haaker et al. (2015)	41	Shapes	Shock	L-DOPA versus placebo	L-DOPA or placebo immediately following extinction	6	1 week	SCR, fMRI, fear ratings
Hagedorn et al. (2022)	60	Shapes	Shock	Hydrocortisone versus placebo, generalization	Hydrocortisone or placebo 40 min prior to extinction, generalization during both extinction and recall	4	24 h	SCR, fMRI
Hauck et al. (2024)	89	Neutral faces	Shock	Glucose versus saccharine (placebo)	After extinction	12	24 h	SCR, FPS, expectancy
(Hofmann et al., 2019)	81	Lamp colours	Shock	DCS versus placebo in SAD	DCS or placebo prior to extinction	8	24 h	SCR, expectancy
Klumpers et al. (2012)	54	Coloured neutral faces	Shock	DCS, THC versus placebo	DCS, THC, or placebo 2 h prior to extinction	8	2 days	SCR, FPS
Kroes et al. (2016)	46	Lamp colours	Shock	Propranolol versus placebo	Propranolol or placebo prior to extinction	12	24 h	SCR, fMRI, fear ratings, explicit memory
Kuriyama et al. (2011)	59	Shapes	Shock	DCS, VPA, DCS + VPA versus placebo, no typical extinction phase	Administered prior to day 2 learning, where a different CS was paired with shock	3	24 h	SCR
Kwee et al. (2024)	69	Neutral faces	Shock	Cannabidiol versus placebo	80 min before retention phase	20	1–2 weeks	SCR, FPS, expectancy, fear ratings^
Luyten et al. (2024)	72	Coloured lamps	Shock	Propranolol versus placebo	60 min before extinction	4	24 h	SCR, FPS, expectancy^
Maples-Keller et al. (2022a), Maples-Keller et al. (2022b)	34	Coloured shapes	Air blast	MDMA versus placebo	Prior to extinction	16	2 days	FPS^
Maples-Keller et al. (2022a), Maples-Keller et al. (2022b)	45	Lamp colours	Loud noise	FAAH inhibition versus placebo, cortisol, AEA, 2-AG, OEA, PEA measured in plasma	FAAH inhibition or placebo for 8 days prior to conditioning. Plasma drawn after extinction and recall	5	24 h	FPS, SCR, heart rate, valence, arousal*
Merz et al. (2018)	40	Lamp colours	Shock	Hydrocortisone versus placebo. Saliva cortisol measured	Hydrocortisone or placebo prior to extinction. Saliva before and after drug administration	8	24 h	SCR, fMRI
Rabinak et al. (2013)	28	Coloured shapes	Loud noise	THC versus placebo	THC or placebo 2 h prior to extinction	20	24 h	SCR, expectancy
Rabinak et al. (2014)	28	Coloured shapes	Loud noise	THC versus placebo	THC or placebo 2 h prior to extinction	20	24 h	SCR, fMRI, expectancy*
Rabinak et al. (2018)	75	Coloured shapes	Loud noise	THC versus placebo	THC or placebo 2 h prior to extinction	20	24 h	fMRI
Schenker et al. (2025)	30	Lamp colours	Shock	Suvorexant, temazepam versus placebo	After extinction, 30 min before sleep	5	48 h	SCR
Sperl et al. (2022)	51	Neutral faces	Loud noise	Yohimbine, sulpiride versus placebo	Between acquisition and extinction	60	24 h	SCR, heart rate, ERP, arousal
Vizeli et al. (2022)	29	Coloured fractals	Scream, air blast	MDMA versus placebo, plasma oxytocin measured	MDMA or placebo 2 h prior to extinction, immediately after acquisition, blood four times after MDMA/ placebo	15	24 h	SCR, FPS
Wen et al. (2021)	90	Lamp colours	Shock	Estradiol versus placebo	Estradiol versus placebo 5 h prior to extinction	8	24 h	SCR, fMRI*
Zabik et al. (2024)	36	Coloured bags	3D virtual snake	5 mg versus 10 mg THC versus placebo	THC or placebo 2 h prior to extinction	20	24 h, 1 week	fMRI, expectancy

*Use of extinction retention index, ^use of correction to extinction learning. 2-AG = 2-arachidonoyl glycerol, AEA, arachidonoyl ethanolamide; DCS, D-cycloserine; fMRI, functional magnetic resonance imaging; FPS, fear potentiated startle; FAAH, fatty acid amide hydrolase; h, hours; L-DOPA, levodopa; MDMA, 3,4-Methylenedioxymethamphetamine; OEA, oleoylethanolamide; PEA, palmitoylethanolamide; SCR, skin conductance response; THC, delta9-tetrahydrocannabinol; VPA, valproate.

However, some effect of L-DOPA on spontaneous recovery have emerged using neuroimaging. Relative to placebo, L-DOPA administered after fear extinction learning resulted in reduced activity in the amygdala and enhanced vmPFC and hippocampal activity during extinction retention, and this same effect was found when delta9-tetrahydrocannabinol was administered too (Haaker et al., 2015; Rabinak et al., 2014). Similarly, propranolol compared to placebo resulted in reduced fear recall, which was accompanied by reduced dorsomedial PFC activity but higher hippocampal activity (Kroes et al., 2016). Cortisol administration prior to extinction retention resulted in higher left insula and amygdala activation in response to stimuli similar to the CS+ and CS−, relative to placebo (Hagedorn et al., 2021). The same group found that cortisol administered prior to extinction training had the opposite effect, with increased vmPFC connectivity to the hippocampus during recall in the active compared to the placebo group (Merz et al., 2018), suggesting that timing of cortisol administration during fear conditioning is critical to its effects on learning and memory. These studies suggest that various pharmacological agents have the potential to modulate relevant neurobiological networks during spontaneous recovery.

The long-held hypothesis that d-cycloserine improves extinction retention has not been supported in human fear conditioning research. Guastella et al. (2007) found over three experiments that there was no effect of 50 mg or 500 mg doses of d-cycloserine on SCRs or US expectancy ratings during extinction retention. These null effects were replicated in three subsequent studies (Ebrahimi et al., 2020; Hofmann et al., 2019; Klumpers et al., 2012). However, d-cycloserine was associated with reduced amygdala and left posterior hippocampus activity in response to the CS+ during retention, *η_p_*^2^ = 0.06–0.08 (Ebrahimi et al., 2020). A similar drug (Yohimbine) also did not result in better extinction retention for valence ratings or SCRs, although analysis of cardiac responses reported some evidence of improved extinction retention relative to placebo (Sperl et al., 2022). Administration of propranolol prior to extinction resulted in successful extinction retention in one study (Kroes et al., 2016), but Luyten et al. (2024) reported no effect.

Studies that administer estradiol or monitor natural estrogen levels have revealed a potential role for estrogen in strengthening extinction. Two translational studies (total *N* = 45) conducted by Graham and Milad (2013) noted that participants who were administered estradiol or had naturally high estrogen levels had significantly lower spontaneous recovery compared to controls. Similarly, administered estradiol resulted in stronger extinction retention compared to placebo in Wen et al. (2021) who compared SCRs between extinguished and non-extinguished CSs. Conversely, Kaczmarczyk et al. (2024) found that estradiol administered prior to extinction resulted in significantly higher spontaneous recovery of SCRs compared to placebo and progesterone controls.

Other studies assessing the effects of exogenous administration of hormones and other compounds have produced mixed results. While cortisol and progesterone have demonstrated little effect on extinction retention of CS+/CS− discrimination compared to placebo or low hormone groups (Hagedorn et al., 2022; Merz et al., 2018; Milad et al., 2010), cannabinoid focused studies have produced more promising results. Mayo et al. (2020b) found that inhibiting fatty acid amide hydrolase (therefore elevating the endocannabinoid anandamide) significantly enhanced extinction retention of SCRs, a finding that has been supported in biomarker analysis of anandamide during extinction retention (Ney et al., 2021a). Direct cannabinoid stimulation via d^9^-tetrahydrocannabinol administration has produced significant SCR extinction retention compared to placebo (Rabinak et al., 2013), though Rabinak et al. (2014) and Klumpers et al. (2012) did not replicate these findings with both treatment and placebo conditions exhibiting successful extinction retention of SCRs at test. Administration of the non-psychoactive cannabinoid cannabidiol did not significantly improve extinction retention of SCRs in two separate studies, though did produce some improvements in US expectancy (Das et al., 2013; Kwee et al., 2024). Despite these inconsistent effects in psychophysiological outcome measures, both THC and cortisol have been reported to increase vmPFC as well as hippocampal activity relative to placebo during extinction and extinction retention (Merz et al., 2018; Rabinak et al., 2014; Rabinak et al., 2018; Zabik et al., 2024).

Effects of psychoactive stimulants have been further explored using other compounds. Methylenedioxymethamphetamine (MDMA) administration resulted in successful extinction retention as demonstrated by SCR, however had no effect on FPS which yielded equivalent fear recall compared to placebo (Vizeli et al., 2022). Maples-Keller et al. (2022a) conversely found that both MDMA and placebo control groups showed significant fear recall and while there was no significant difference when analysed using analysis of variance, there was some evidence of proportionally more participants from the MDMA group demonstrating extinction retention compared to the placebo group. Similarly, oxytocin delivered prior to extinction learning increased extinction retention relative to placebo (Acheson et al., 2013). No effect of suvorexant or temazepam was observed on extinction retention in Schenker et al. (2025) though a study using caffeine as an intervention found that at retention only the placebo group exhibited spontaneous recovery of fear in SCRs, *η_p_^2^* = 0.23 (Culver et al., 2014). Finally, one study found that glucose, administered after extinction showed some evidence of reducing spontaneous recovery in FPS, but not SCR or US expectancy (Hauck et al., 2024).

In summary, these studies show that testing spontaneous recovery during fear conditioning can assist in the identification of pharmaceuticals that may improve memory mechanisms thought to underlie psychiatric disorders such as anxiety and PTSD (Abraham et al., 2014; Glover et al., 2015; Hwang et al., 2015; Ney et al., 2022a). From the perspective of establishing the paradigm’s validity, it is promising that compounds such as d-cycloserine, which have produced null effects in spontaneous recovery experiments, have similarly shown a lack of positive outcomes in clinical trials (McGuire et al., 2017). However, timing of drug administration has varied significantly across studies (see Table 3), and there is no consensus on when drugs should be delivered for effective consolidation of extinction memories. The spontaneous recovery paradigm should be further investigated as a potential approach to fast-fail test novel therapeutics for PTSD and anxiety disorders.

**Table:** 

Summary—pharmaceutical interventions
Emerging evidence for estradiol, caffeine, propranolol, and oxytocin interventions on spontaneous recovery
Mixed evidence for cannabinoid, MDMA, and L-DOPA interventions on spontaneous recovery
Weak evidence for cortisol and d-cycloserine
Timing of pharmaceutical intervention relative to extinction is inconsistent across studies and it is unclear how this should effectively translate to clinical interventions during exposure

### Augmenting fear extinction memories with non-pharmacological methods

Researchers have explored how the extinction phase can be enhanced using non-pharmacological methods to elicit stronger extinction retention, which may lead to the development of more effective psychological therapies (Table 4).

**Table 4:** Use of spontaneous recovery paradigms with non-pharmacological interventions.

Study	Sample size	CS	US	Manipulation	Manipulation timing	Trials during recall	Extinction—recall delay	Outcome measures
An and Chen (2016)	30	Yellow and blue squares	Loud noise	Time of recall (within-subjects)	n/a	10	1- and 30-day recall tests	SCR
Antov et al. (2015)	40	Shapes	Loud noise	Stress induction versus control	Stress induction or control prior to extinction	12	24 h	SCR*
Asthana et al. (2016)	91	Blue and yellow squares	Scream	Reactivation versus standard extinction, val66met genotyping	Reactivation prior to extinction	16	24 h	SCR^
Bierwirth et al. (2023)	40	Neutral faces	Loud noise	Immediate versus delayed extinction	Extinction either 10 min or 24 h following acquisition	60	24 h	SCR, EEG, heart rate, valence, arousal
Björkstrand et al. (2019)	26	Blue or purple squares	Shock	Mindfulness versus waitlist	Mindfulness or waitlist completed prior to fear conditioning	10	24 h	SCR^
Björkstrand et al. (2026)	55	Coloured shapes	Shock	Mindfulness versus waitlist	Mindfulness or waitlist completed prior to fear conditioning	4	24 h	SCR, fMRI
Brown et al. (2025)	24	Abstract symbols	Shock	Deepened extinction	After standard extinction	8	7 days	SCR
Boukezzi et al. (2017)	18	Shapes	Shock	Alternating bilateral stimulations	During extinction	48	24 h	SCR, expectancy
Carpentier et al. (2024)	74	Animals	Shock	Addition of approach behaviour (zooming in to CSs)	During extinction	4	24 h	SCR, expectancy, valence
Chalkia et al. (2020)	124	Yellow and blue squares	Shock	Reactivation.	Reactivation 10 min prior to extinction	10	24 h	SCR^
Chen et al. (2021a)	84	Shapes or neutral sounds	Shock	Reactivation with reinforced or unreinforced CS+	Reactivation 10 min prior to extinction	10	24 h	SCR^
Chen et al. (2021a)	66	Shapes	Shock	Reactivation.	Reactivation 10 min prior to extinction	6	24 h	SCR, FPS
Chen et al. (2022)	64	Red or yellow squares	Shock	Standard extinction versus NFE or counterconditioning	During extinction	11	24 h	SCR
Coelho et al. (2015)	51	Blue or green squares	Shock	Deepened extinction versus control	During extinction	12	24 h	SCR, expectancy^
Culver et al. (2018)	39	Neutral faces	Scream	Partial reinforcement extinction versus control	¼ of trials reinforced during extinction	4	1 week	SCR, expectancy, valence^
Dar and Asthana (2025)	63	Neutral faces	Scream	Reappraisal versus standard extinction	During extinction	6	24 h	SCR, expectancy, valence
Dar and Asthana (2026)	51	Neutral faces	Scream	Inhibitory control versus standard extinction	During extinction	6	24 h	SCR, expectancy, valence, arousal
Dunsmoor et al. (2014)	43	Virtual characters	Shock	Extinction conducted in acquisition, extinction, or multiple contexts	During extinction	4	24 h	FPS
Dunsmoor et al. (2019)	46	Angry faces	Shock	Novelty facilitated extinction versus standard extinction	During extinction	12	24 h	SCR, fMRI^
Dural et al. (2022)	219	Snakes and spiders, coloured circles	Shock	Time of recall (within-subjects, type of CS, reconsolidation reminder)	Reconsolidation reminder: 10 min or 6 h prior to extinction, or no reminder	11	24 h, 15 days, and 3 months	SCR, arousal^
Feng et al. (2016)	40	Coloured squares	Pictures	Reactivation versus no reactivation	10 min prior to extinction	60	24 h	fMRI, fear ratings*
Hamacher-Dang et al. (2015)	40	Coloured lamps	Shock	Stress versus control. Context manipulated, confounds recall	Stress or control administered after extinction	5	24 h	SCR, expectancy
Hartley et al. (2014)	59, 34	Coloured shapes	Shock	Escapable stress, inescapable stress versus control	Before (experiment 1 and after) (experiment 2) fear conditioning	4	24 h	SCR*^
Hartley et al. (2019)	48	Fearful faces	Shock	Active avoidance versus yoked partially reinforced extinction	During extinction	10	24 h	SCR, blink rate*
Huff et al. (2009)	66	Snakes, spiders	Shock	Immediate versus delayed extinction, context shift versus no context shift	Context shift during extinction. Delayed extinction delayed 24 h	16	24 h	SCR^
Javanbakht et al. (2017)	40	Coloured lamps	Loud noise	Instructed acquisition versus instructed extinction versus instructed extinction and recall	Instructions provided either prior to acquisition, extinction, or extinction and recall	15	24 h	SCR
Jiang and Greening (2021)	56	Letters	Shock	Imagined versus visual CS	Imagined or visual CS during extinction	8	Not reported	SCR, fear ratings, expectancy
Junjiao et al. (2021)	50	Coloured shapes	Shock	Reactivation. Stress versus control	Reactivation 10 min prior to extinction. Stress or control after reactivation	8	24 h	SCR, FPS
Kang et al. (2018)	40	Spiders	Shock, positive cartoon images	Counterconditioning versus standard extinction	During extinction	12	24 h	Expectancy, arousal^
Kindt and Soeter (2013)	40	Spiders	Shock	Reactivation and extinction versus extinction only	Reactivation 10 min prior to extinction	12	24 h	SCR, FPS, expectancy^
Kitamura et al. (2022)	75	Coloured squares	Shock	Reinforcement rate 40% or 80%. Reactivation.	Reinforcement during acquisition. Reactivation 10 min prior to extinction	6	24 h	SCR^
Laing and Dunsmoor (2026)	112	Animals, tools (categories)	Shock	NFE versus counterconditioning versus standard extinction	During extinction	6	24 h	SCR, valence, fear ratings*
Li et al. (2017)	80	Coloured shapes (compound CS)	Shock	No reactivation versus reactivation with 1/3, 2/3, or all of the compound CS	10 min prior to extinction	8	24 h	SCR^
Ma et al. (2025)	120	Coloured lamps	Shock	Number and timing of extinction trials varied	During extinction	8	24 h, 1 week	SCR, valence, arousal, fear ratings
Meir Drexler et al. (2014)	39	Aversive animal pictures	Shock	Context manipulation. Reactivation	Reactivation 10 min prior to extinction	8	24 h	SCR, expectancy
Ney et al. (2023a), Ney et al. (2023b), Ney et al. (2023c)	110, 109	Words	Loud noise	Level of processing of words: high, low versus none	During extinction	8, 16	48 h	Reaction time, valence, expectancy
Norrholm et al. (2008)	92	Coloured light	Air blast	Delayed (72 h versus immediate extinction, single cue versus differential conditioning)	n/a	4	24 h, 4 days	FPS, expectancy^
Oyarzún et al. (2019)	35	Fearful faces	Shock	Implicit versus explicit extinction	During extinction	6	24 h	SCR, FPS, expectancy^
Pace-Schott et al. (2013)	109	Coloured lamps	Shock	Time of day and duration between extinction and recall. Actigraphy and sleep timing measured.	Sessions started in morning or evening, and recall started 3, 12, or 24 h after extinction	8	3 h, 12 h, 24 h	SCR, expectancy
Pace-Schott et al. (2015)	109	Coloured lamps	Shock	Time of day and duration between extinction and recall	Sessions started in morning or evening, and recall started 3, 12, or 24 h after extinction	8	3 h, 12 h, 24 h	SCR, expectancy
Pan et al. (2024)	78	Birds, fish	Shock	CS or US reactivation versus standard extinction	Prior to extinction	3	24 h	SCR, EEG
Pappens et al. (2015)	39	Shapes	Breathing occlusion	Generalization	During extinction	4	24 h	SCR, FPS, respiration, expectancy^
Quezada-Scholz et al. (2018)	47	Shapes	Shock	Extinction cue presented during extinction	n/a	6	2 days	SCR, expectancy
Quintero et al. (2024)	157, 107	Coloured shapes	Loud noise	Occasionally reinforced extinction versus standard extinction	During extinction	10	6 min, 24 h	Expectancy^
Raio et al. (2014)	52	Fractal geometry	Shock	Stress induction versus control	Stress induction or control prior to recall	10	24 h	SCR^
Scheuermann et al. (2025)	35	Animal, tools, food categories	Shock	Within subjects: Memory updating	After extinction	16	24 h	FPS, SCR, expectancy, fear ratings
Schiller et al. (2010)	65	Yellow and blue squares	Shock	Reactivation versus no reactivation of CS	10 min and 6 h prior to extinction	10	24 h	SCR^
Sevinc et al. (2019)	67	Coloured lamps	Shock	Mindfulness training	8 weeks before fear conditioning	8	24 h	fMRI*
Shi et al. (2018b), Shi et al. (2018a), Shi et al. (2018c)	125	Red and yellow squares	Shock	Fasting versus control	Prior to extinction or acquisition	20	24 h, 6 months	SCR
Thompson and Lipp (2017)	56	Spider, snake, blue and yellow squares	Shock	US reactivation versus no reactivation	10 min prior to extinction	8	24 h	SCR, valence^
Thompson et al. (2018)	72	Birds, fish	Shock	US presented unpaired to CS, partially reinforced to CSs, or standard extinction	During extinction	6	10 min	SCR, valence
van Dis et al. (2019)	87	Neutral faces	Shock, funny film clips	Counter- conditioning versus extinction versus non-contingent positive film clips	After acquisition	2	7 days	SCR, FPS, valence, arousal, fear ratings^
Warren et al. (2014)	55	Shapes	Air blast	Reactivation versus no reactivation of CS	Reactivation 10 min prior to extinction	4	24 h	FPS, expectancy^
Waters et al. (2022)	62	Shapes	Loud noise	Liked music versus no music	Music or no music presented during extinction	3	10 min	Expectancy, arousal^
Wendt et al. (2020)	112	Neutral faces	Shock	Instructed extinction versus instructed extinction with electrode removed versus standard extinction	During extinction	4	24 h	SCR, FPS, valence
Wolsink et al. (2026)	120	Lamp colours	Shock	Psychosocial stress versus exercise versus control	Prior to extinction	4	24 h	SCR, pupil size,
Yang et al. (2019)	36	Coloured shapes	Shock, money reward	Reactivation. One group has CS+ reinforced both by shock and money reward	Reactivation prior to extinction	6	24 h	SCR, FPS*^
Yoshiike et al. (2018)	29	Shapes	Shock	High versus low (control intensity blue light)	High or low intensity light during extinction	3	24 h	SCR, fNIRS
Zbozinek et al. (2015)	84	Coloured circles	Shock	Number of CSs presented and CS duration during extinction	During extinction	2	24 h	Fear ratings, valence^
Zbozinek (2017)	58	Neutral faces	Shock	Negative or positive mood induction	Prior to extinction and in between extinction phases	2	1 week	SCR, FPS, expectancy, fear ratings*
Zhuang et al. (2017)	37	Coloured shapes, sounds	Shock	Visual versus auditory CS	During extinction	10	24 h	SCR^

*Use of extinction retention index, ^use of correction to extinction learning. CS, conditioned stimuli; EEG, electroencephalography; fMRI, functional magnetic resonance imaging; fNIRS, functional near-infrared spectroscopy; FPS, fear potentiated startle; h, hours; min, minutes; NFE, novelty facilitated extinction; OCD, obsessive compulsive disorder; PTSD, posttraumatic stress disorder; SCR, skin conductance response; US,unconditioned stimulus.

The idea that fear memories entered a labile state during reconsolidation was first explored by Schiller et al. (2010), who reported that a single unreinforced CS+ presented 10 min prior to extinction improved extinction retention as measured by SCR; however, this finding has been replicated in only a minority of subsequent studies (Dural et al., 2022; Kitamura et al., 2022). Kindt and Soeter (2013) employed fear-relevant stimuli (spiders) during fear conditioning and recorded spontaneous recovery across SCR, FPS, and US expectancy. No effect of memory reactivation was found on spontaneous recovery. Likewise, Chalkia et al. (2020) used a robust sample size (*N* = 244) and nevertheless reported significant return of fear for all participants, irrespective of whether they experienced unreinforced CS+ presentation prior to extinction. Li et al. (2017) and Yang et al. (2019) provided further evidence against the hypothesis that fear memory reactivation prior to extinction reduces fear recall, with spontaneous recovery occurring for multiple CS+s exposed to various conditions across their study designs (*η^2^* = 0.042). Warren et al. (2014), Pan et al. (2024), and Meir Drexler et al. (2014) were also unable to replicate the memory reactivation effect. Stronger functional coupling between the amygdala and vmPFC was evident during extinction retention in a group that underwent standard extinction training compared to a group that received a fear reminder and reconsolidation period prior to extinction training (Feng et al., 2016).

Chen et al. (2021b) tested an alternate paradigm that sought to leverage prediction error effects on extinction retention. In their first study, FPS and SCR data revealed that prediction error led to extinction retention only when acquisition shock schedule was predictable. In the second experiment both groups experienced unpredictable shocks and were provided either one or two prediction error interventions. Both groups recorded extinction retention. Junjiao et al. (2021) replicated Chen et al.’s design with an added stressor test between acquisition and extinction. They reported SCR extinction retention only for those with predictable US schedule and no stress test, but high fear recall in FPS for all groups. The negative effect of stress on extinction retention was similarly replicated by Raio et al. (2014), Hamacher-Dang et al. (2015), and Hartley et al. (2014) though a purely physiological stressor prior to extinction improved extinction retention Antov et al. (2015) and Peyrot et al. (2024) as well as Wolsink et al. (2026) found no effect of psychosocial stress induction prior to extinction on spontaneous recovery. A final study from this group found that two, but not one or four, CS+ presentations prior to extinction decreased spontaneous recovery, suggesting that this prediction error effect may be affected by the number of CS+ presentations during memory reconsolidation (Chen et al., 2022). Similarly, Zhuang et al. (2017) found that reactivation of only the tone component of an auditory tone-visual shape compound CS+ reduced spontaneous recovery of fear, suggesting that the effect predicted by Schiller et al. (2010) may be confined to strict boundary conditions to reliably replicate.

Studies that have sought to augment extinction learning in other ways have also reported improvements in extinction retention. Thompson and Lipp (2017) reported that an unsignalled US presented at half its original intensity prior to extinction training led to extinction retention compared to controls (*η_p_^2^* = 0.09). Incorporating the US as part of an extinction phase when both unpaired to the CS+ and randomly paired with the CS+ has also produced significantly improved extinction retention compared to standard extinction when measured via SCR, *η_p_^2^* = 0.09 (Thompson et al., 2018). The concept of ‘deepened extinction’ has also been explored, which is when extinction of multiple (compared to single) CS+s presented during acquisition leads to stronger extinction retention, though results have been mixed (Brown et al., 2025; Coelho et al., 2015). Participants exposed to massed extinction—compared to multiple, smaller, and temporally distinct extinction phases—had significantly better extinction retention (Ma et al., 2025).

US expectancy and SCRs show extinction retention following generalised extinction, where only one of a range of similar CS+s is extinguished during extinction learning (Pappens et al., 2015). Moreover, participants exposed to an active avoidance condition, where they felt in control of the number of USs delivered during extinction, had significantly reduced spontaneous recovery compared to participants in a standard extinction group (Hartley et al., 2019), though approach behaviour during extinction (zooming into the CS) did not reduce spontaneous recovery (Carpentier et al., 2024), nor did CS reappraisal, when compared to standard extinction (Dar and Asthana, 2025). Dunsmoor et al. (2019) observed lower differential vmPFC activity during extinction retention in participants that completed novelty-facilitated compared to standard extinction.

As therapeutic intervention typically occurs in the novel context of a therapist’s office, studies have also sought to understand how different learning contexts impact learned associations. Participants who completed a multiple-context extinction phase, a consistent context across phases, or a single novel context during extinction all experienced spontaneous recovery of fear as measured by FPS (Dunsmoor et al., 2014). Huff et al. (2009) also observed that context change (relative to no context change) had no effect on 24-h extinction retention if the extinction phase occurred 5 min after acquisition, but when extinction occurred 24 h post-acquisition, extinction retention was observed for those who experienced no context change, while spontaneous recovery of SCR occurred for those who experienced the novel extinction context. Therefore, context change seems to affect 24-h extinction retention only under certain conditions like sufficient time in between phases, and potentially if assessed using SCR.

Several innovative interventions with potentially direct therapeutic applications have been successfully tested during extinction. Dorsomedial PFC activity was reduced during retention in participants who completed an exercise manipulation during extinction compared to extinction-only controls (Zhou et al., 2025). Boukezzi et al. (2017) reported that bilateral auditory stimulation during extinction learning reduced spontaneous recovery of conditioned fear (measured by SCRs but not US expectancy—suggesting a subconscious effect), while Oyarzún et al. (2019) found spontaneous recovery was present regardless of whether CS+s were consciously and unconsciously perceived during extinction learning. Interestingly, de Voogd et al. (2018) reported higher dACC activity in response to the CS+ compared to CS− during extinction retention in a control group compared to rapid eye movement intervention (de Voogd et al., 2018). Kang et al. (2018) and Wirz et al. (2024) found that counterconditioning, in place of standard extinction, significantly reduced spontaneous recovery, though this was not replicated in Quintero et al. (2024), van Dis et al. (2019) or Laing and Dunsmoor (2026), the latter of whom found that novelty-facilitated extinction performed better than counterconditioning. Similarly, Zbozinek et al. (2015) found that post-extinction CS+ valence predicted the level of spontaneous recovery, but positive mood induction following acquisition did not improve extinction retention in a subsequent study (Zbozinek, 2017). Instructional extinction successfully reduced spontaneous recovery when compared to instructions of CS+/US contingency given to participants prior to acquisition, *η*^2^ = 0.13 (Javanbakht et al., 2017), though this effect was not replicated in a subsequent study, *R^2^* = 0.027 (Wendt et al., 2020).

While positive mood induction and counterconditioning were unsuccessful, Waters et al. (2022) reported that verbalisation of a phrase “look and learn” prior to reporting trial-by-trial CS-US contingencies during extinction reduced spontaneous recovery during the extinction test (*η_p_^2^* = 0.13), Scheuermann et al. (2025) found that memory updating (by asking participants to imagine engaging with stimuli similar to the CS+) also reduced spontaneous recovery, and Dar and Asthana (2026) subsequently reported that spontaneous recovery was reduced in participants who completed inhibitory training (stop signal task) during extinction compared to standard extinction. Moreover, Jiang (2021) found that combining deliberate mental imagery of CSs with extinction training improved extinction retention (Jiang and Greening, 2021). This suggests that actively engaging participants in tasks that train inhibition or attention towards CSs during extinction may improve extinction retention.

Interestingly, 16 h of fasting prior to fear conditioning did not affect fear acquisition but did improve extinction retention (Shi et al., 2018c). The time of day at which extinction learning is completed has also been reported to affect subsequent extinction retention, with Pace-Schott et al. (2013) and Pace-Schott et al. (2015) finding that spontaneous recovery of fear is significantly reduced if extinction learning is completed in the morning compared to the evening. Gong et al. (2025) found that social support during extinction training improved retention. Another interesting intervention was explored in Yoshiike et al. (2018) where the room lights were either bright or normal lighting during extinction learning. Bright lighting was found to reduce spontaneous recovery (Yoshiike et al., 2018). Finally, Björkstrand et al. (2019) found that participants who completed mindfulness training via a smartphone app for 4 weeks prior to undergoing fear acquisition had significantly improved extinction retention compared to waitlist controls (*d* = 0.98) and η^2^ = 0.088 for a subsequent study (Björkstrand et al., 2026). Reductions in spontaneous recovery as a result of mindfulness training were found to be correlated with hippocampal activity using fMRI (Leibovitz et al., 2024; Sevinc et al., 2020; Sevinc et al., 2019).

In summary, the effect of various non-pharmacological interventions administered with the intention of altering fear and/or extinction memories has been tested in spontaneous recovery paradigms. Memory reconsolidation has become controversial as few studies have been able to replicate the phenomenon. Augmentation of extinction learning via various other interventions may show more promise, as these interventions have routinely been shown to reduce spontaneous recovery, particularly those that involve deliberate participant involvement. However, to our knowledge, when compared to pharmacological interventions, fewer of these interventions have been trialled in clinical settings. This section highlights that there are few attempts to directly replicate interventions using the spontaneous recovery paradigm, as most studies either used completely novel paradigms, or implemented existing interventions without direct replication (i.e., introduction of competing factors).

**Table:** 

Summary—augmenting fear extinction memories
Memory reactivation prior to extinction was popular but has repeatedly failed to replicate in subsequent studies
Many other interventions during extinction have emerged, with successful options including deepened extinction, active avoidance, presentation of unpaired USs, bilateral auditory stimulation, fasting, extinction in the morning, bright light, mental imagery of CSs, CS understanding, and mindfulness
Contrary to those in the pharmacological section, most interventions arising from this literature have not progressed to clinical trials

### Augmenting extinction memories with sleep

Nineteen studies have tested the effect of sleep on retention of extinction memories (Table 5). Following Schiller et al. (2010), one study tested the effect of re-exposure to a neutral non-CS tone that had been presented during extinction either during slow wave sleep or while awake, finding that re-exposure to the extinction tone during slow wave sleep increased spontaneous recovery (Ai et al., 2015). Chen et al. (2021a) similarly found that presentation of a retrieval cue prior to extinction learning reduced spontaneous recovery if recovery occurred either before or after a night’s sleep.

**Table 5:** Use of spontaneous recovery paradigms with measurement of sleep.

Study	Sample size	CS	US	Manipulation	Manipulation timing	Trials during recall	Extinction—recall delay	Outcome measures
Ai et al. (2015)	133	Blue or yellow squares	Shock	Sleep versus wake, reactivation versus no reactivation	After extinction	5	3 h	SCR^
Bottary et al. (2020)	48	Lamp colours	Shock	Insomnia versus good sleepers. Actigraphy and PSG measured.	Actigraphy measured 2 weeks prior to conditioning. PSG measured prior to conditioning	8	24 h	SCR, expectancy*
Chen et al. (2021a)	96	Coloured shapes	Shock	Reactivation. 12 h sleep versus 12 h no sleep versus 3 h break	Reactivation prior to extinction. Sleep/break / no sleep after extinction	6	3 h, 12 h, 24 h	SCR, expectancy^
Friesen et al. (2022)	51	Neutral faces	Film clips	Sleep versus wake. PSG recorded	Sleep or wake prior to extinction learning	6	4 h	SCR, FPS, fear ratings, expectancy^
Friesen et al. (2023)	107, 74	Neutral objects	Film clips	Hypnotic suggestion versus control text. PSG recorded	Hypnotic suggestion or control text after extinction (performed overnight during sleep)	8	24 h	SCR, expectancy
Lucifora et al. (2022)	40	Blue and red door (virtual reality)	Monster + scream	Intermediate versus evening chronotype. Sessions conducted in either evening or morning	After extinction learning.	10	24 h	SCR, fear ratings
Menz et al. (2016)	80	Shapes	Shock	Sleep manipulated—no sleep versus REM sleep versus SWS sleep. PSG measured.	After extinction learning, split night study.	10	2 days	SCR, fMRI^
Pace-Schott et al. (2009)	53	Coloured lamps	Shock	Sleep versus wake.	Extinction conducted in the morning or night	8	12 h	SCR, expectancy
Pace-Schott et al. (2014)	28	Coloured lamps	Shock	Time of day and duration between extinction and recall. PSG measured	Sessions started in morning or evening, and recall started 3, 12, or 24 h after extinction	8	3 h, 12 h, 24 h	SCR, FPS, heart rate, expectancy*
Pace-Schott et al. (2015)	109	Coloured lamps	Shock	Time of day and duration between extinction and recall	Sessions started in morning or evening, and recall started 3, 12, or 24 h after extinction	8	3 h, 12 h, 24 h	SCR, expectancy
Schenker et al. (2022)	248	Coloured circles	Shock	PTSD versus trauma and non-trauma control. Sleep quality measured using sleep diaries and PSQI	n/a	10	2 days	SCR^
Seo et al. (2018)	46	Lamp colours	Shock	Insomnia versus good sleepers	PSG measured prior to conditioning	8	24 h	SCR, fMRI
Seo et al. (2022)	126	Lamp colours	Shock	PTSD versus trauma controls	PSG measured prior to conditioning and after extinction	8	24 h	SCR, fMRI, expectancy
Spoormaker et al. (2010)	16	Shapes	Shock	Afternoon nap with or without REM sleep. PSG measured	Nap after extinction	15	4 h	SCR, fMRI
Spoormaker et al. (2012)	18	Shapes	Shock	REM sleep versus non-REM sleep deprivation	Night after extinction learning	15	24 h	SCR, fMRI^
Straus et al. (2017)	71	Coloured circles	Shock	Normal sleep versus 36 h sleep deprivation	Sleep deprivation either before or after extinction	8	24 h	FPS, anxiety ratings, expectancy
Straus et al. (2018)	13	Coloured circles	Air blast	Veterans with PTSD, PSG measured	PSG measured before and after extinction	8	24 h	FPS*
Vanuk et al. (2022)	82	Coloured cars	Shock	PTSD, blue light treatment	Blue light each morning 6 weeks prior to recall	16	6 weeks	SCR, fMRI, heart rate*
Yuksel et al. (2024)	113	Lamp colours	Shock	PSG recorded as well as sleep and nightmare diaries	PSG measured before and after extinction session	8	24 h	SCR*

*Use of extinction retention index, ^use of correction to extinction learning. fMRI, functional magnetic resonance imaging; FPS, fear potentiated startle; h, hours; PSG, polysomnography; PSQI, Pittsburgh Sleep Quality Inventory; PTSD, posttraumatic stress disorder; REM, rapid eye movement; SCR, skin conductance response; SWS, slow wave sleep.

While the direct effect of sleep on spontaneous recovery has been assessed, findings are inconsistent. Participants with insomnia tend to show higher spontaneous recovery compared to good sleepers (Bottary et al., 2020; Seo et al., 2018) and there is one report of acute sleep deprivation reducing extinction retention, *d* = 0.65 (Straus et al., 2017), though Pace-Schott et al. (2009) reported no effect of overnight sleep (versus daytime wakefulness) on extinguished SCRs towards CSs during a subsequent extinction test, Lucifora et al. (2022) found no effect of evening chronotype on extinction retention of SCRs or fear ratings, and Friesen et al. (2022) found no effect of early night sleep (versus wakefulness) on US expectancy or fear ratings during extinction retention. This result is consistent with previous research that found no effect of early night sleep versus wakefulness on extinction retention using US expectancy ratings and SCRs (Menz et al., 2016), though this study did find that late night sleep resulted in significantly better extinction retention compared to late night wakefulness. Friesen et al. (2023) reported no effect of sleep hypnosis (an audio recording of hypnotic trance induction and a suggestion to sleep deeper) on spontaneous recovery, even though hypnosis resulted in better quality sleep. Finally, two studies have found that subjectively reported measures of higher sleep efficiency, as measured by the Pittsburgh Sleep Quality Index and the ratio of time sleeping to time spent in bed, are correlated with improved extinction retention (Pace-Schott et al., 2015; Schenker et al., 2022), though the latter study only found this effect in women but not men.

The idea that longer duration and higher quality of rapid eye movement (REM) sleep affects extinction memory consolidation and subsequent extinction retention has been supported by multiple studies of healthy participants in the literature (Bottary et al., 2020; Pace-Schott et al., 2014; Spoormaker et al., 2012; Spoormaker et al., 2010; Straus et al., 2018; Yuksel et al., 2024), though Bottary et al. (2020) found that longer REM sleep was correlated with higher spontaneous recovery in participants with insomnia disorder.

Overall, the evidence for a direct effect of sleep on spontaneous recovery is inconsistent, with some studies finding no significant impact of sleep deprivation or early night sleep on extinction retention. However, some studies have reported that REM sleep appears to improve extinction retention.

**Table:** 

Summary—effect of sleep
Sleep deprivation studies have not consistently reported significant effects of post-extinction sleep on extinction retention
However, REM sleep appears to facilitate extinction retention and reduce spontaneous recovery

### Brain and vagal nerve stimulation

Non-invasive brain stimulation methods such as transcranial direct current stimulation (tDCS) and transcranial magnetic stimulation (TMS) have been explored as potential methods of improving fear extinction retention (Table 6). Abend et al. (2016) found that participants who received tDCS targeting the medial prefrontal cortex (mPFC) during extinction learning did not show spontaneous recovery as measured by SCRs, whereas spontaneous recovery was observed in both sham and anodal tDCS groups (n = 15 each). Raij et al. (2018) used repetitive TMS over the ventromedial prefrontal cortex (vmPFC) during extinction and found that this reduced return of fear relative to stimulation of a control area. This effect was replicated by Borgomaneri et al. (2020). However, tDCS over the vmPFC during extinction failed to affect spontaneous recovery (Van’t Wout et al., 2016), and cerebellar theta band transcranial alternating current stimulation at 6 hertz also did not affect spontaneous recovery (Schellen et al., 2024). tDCS over the left inferior frontal gyrus during extinction did modulate extinction retention, with anodal stimulation increasing spontaneous recovery of SCRs and cathodal stimulation increasing extinction retention (Ma et al., 2024).

**Table 6:** Use of spontaneous recovery paradigms with brain stimulation.

Study	Sample size	CS	US	Manipulation	Manipulation timing	Trials during recall	Extinction—recall delay	Outcome measures
Abend et al. (2016)	45	Neutral faces	Scream	Direct current, alternating current versus sham	Applied during extinction	8	24 h	SCR, fear ratings
Borgomaneri et al. (2020)	84	Coloured rooms	Shock	Reactivation versus no reactivation. rTMS versus sham	rTMS or sham 10 min after reactivation	4	24 h	SCR, contingency ratings
Burger et al. (2017)	42	Shapes	Shock	tVNS versus sham	tVNS or sham during extinction	6	24 h	SCR, FPS, heart rate, expectancy
Cybinski et al. (2025)		Neutral faces	Scream	iTBS to left PFC	iTBS or sham before extinction	6	24 h	SCR, arousal, valence^
Deng et al. (2021)	70	Coloured squares	Shock	iTBS to vertex versus iTBS to left dlPFC	Before and after extinction	15	24 h, 1 month	SCR^
Guhn et al. (2014)	85	Neutral faces	Scream	rTMS versus sham	Before extinction	20	24 h	SCR, FPS, fNIRS, valence, arousal^
Ma et al. (2024)	177	Coloured lamps	Shock	Anodal versus cathodal tDCS versus sham over left inferior frontal gyrus	During extinction	8	24 h	SCR, valence, arousal, fear ratings
Ney et al. (2021a), Ney et al. (2021b), Ney et al. (2021c)	30	Green and orange circles	Shock	Anodal tDCS versus sham	After extinction	10	24 h	SCR
Raij et al. (2018)	28	Coloured lamps	Shock	rTMS to vmPFC versus rTMS to control	During extinction	4	24 h	SCR
Schellen et al. (2024)	37	Coloured lamps	Shock	6 Hz ctACS versus sham	During extinction	12	24 h	SCR
Su et al. (2022)	170	Coloured squares	Shock	cTBS versus sham. Reactivation versus no reactivation.	cTBS applied either immediately or 6 h after reactivation. Recall completed either after sleep or sleep deprivation	3	12 h, 24 h	SCR^
Szeska et al. (2020)	40	Neutral faces	Shock	Anodal tDCS versus sham	tDCS or sham during first half of extinction	10	24 h	SCR, FPS, heart rate, expectancy
Szeska et al. (2020)	80	Single cue—coloured pentagon	Shock	tVNS versus sham stimulation, reinstatement test	Reinstatement after first recall test. tVNS/sham during extinction	16	20–28 h and then 28 days	FPS, expectancy
Van’t Wout et al. (2016)	44	Coloured lamps	Shock	tDCS with no control	during first half of extinction versus during second half of extinction	8	24 h	SCR
Van't Wout et al. (2017)	28	Coloured lamps	Shock	tDCS with no control. All participants had PTSD	During extinction or after extinction	8	24 h	SCR
Vicario et al. (2020)	32	Green and orange circles	Shock	Anodal tDCS versus sham	tDCS during extinction	10	24 h	SCR

*Use of extinction retention index, ^use of correction to extinction learning. ctACS, cerebellar theta band transcranial alternating current stimulation; cTBS, continuous theta burst stimulation; dlPFC, dorsolateral prefrontal cortex; FPS, fear potentiated startle; h, hours; iTBS, intermittent theta burst stimulation; rTMS, repetitive transcranial magnetic stimulation; SCR, skin conductance response; tDCS, transcranial direct current stimulation; tVNS, Transcutaneous Vagus Nerve Stimulation; vmPFC, ventromedial prefrontal cortex.

Burger et al. (2017) found that, relative to sham, no significant effects of transcutaneous vagal nerve stimulation during extinction were observed on spontaneous recovery indexed by SCR or FPS (*n* = 21 per group). In contrast, Szeska et al. (2020) found that transcutaneous vagal nerve stimulation during extinction resulted in significantly reduced spontaneous recovery (measured by FPS) both 24 h and 28 days following extinction (*η_p_*^2^ = 0.06). Theta-burst TMS over the right dorsolateral prefrontal cortex (dlPFC) was also associated with reduced spontaneous recovery 24 h after a memory reactivation phase (Su et al., 2022). However, this effect was only present if stimulation was applied 10 min but not 6 h following memory reactivation (Su et al., 2022).

The issue of stimulation timing has been explored in this literature, though findings are mixed. It was recently reported that tDCS over the dlPFC directly after fear acquisition/before extinction training (both conducted on day 1) resulted in higher return of fear compared to sham in a 24 h extinction retest (Ney et al., 2021c), though tDCS over dlPFC reduced return of fear when administered after extinction using the exact same paradigm, *η_p_^2^* = 0.238 (Vicario et al., 2020). Van't Wout et al. (2017) similarly reported that, in veterans, tDCS over the vmPFC following extinction training reduced 24-h spontaneous recovery to a greater extent than stimulation presented during extinction (*d =* 0.38). In contrast, Deng et al. (2021) found that theta burst TMS both before and after extinction training over the left dlPFC reduced return of fear in a retention phase 24 h later. Moreover, Guhn et al. (2014) reported that repetitive TMS over the medial PFC after acquisition and before extinction learning (both day 1) resulted in reduced return of fear in a 24 h extinction test (Guhn et al., 2014), but theta-burst TMS applied to the left PFC before extinction had no effect on spontaneous recovery (Cybinski et al., 2025).

**Table:** 

Summary—stimulating the brain
tDCS and TMS have mixed effects on extinction retention
Stimulation of the exact correct location is important—over the dlPFC, mPFC, or vmPFC
Mixed evidence for transcranial vagal nerve stimulation
Timing of stimulation is critical. Research has not yet determined the correct timing to apply stimulation relative to extinction

### Neurotransmitters

The effect of neurotransmitters on spontaneous recovery has been a topic of growing interest as it might provide information about both the mechanisms of spontaneous recovery as well as potential treatment targets (Table 7). Women using hormonal contraceptives are also reported to have higher spontaneous recovery compared to naturally cycling women (Bierwirth et al., 2021; Davignon et al., 2025; Graham and Milad, 2013). This effect is likely due to the negative impact of low estradiol levels on extinction retention—as well as reinstatement—which has been replicated across a number of studies measuring both estradiol as well as controlling for menstrual cycle phases where estradiol levels are naturally reduced, *η_p_*^2^ = 0.09 (Antov and Stockhorst, 2014; Davignon et al., 2025; Felmingham et al., 2021; Li and Graham, 2016; Milad et al., 2006; Milad et al., 2010; Peyrot et al., 2024; White and Graham, 2016; Zeidan et al., 2011). However, while Milligan-Saville and Graham (Milligan-Saville and Graham, 2016) found that estradiol predicted extinction retention in women with no children, this correlation was not found in women with children.

**Table 7:** Use of spontaneous recovery paradigms with measurement and control of neurotransmitters and neurotransmitter systems.

Study	Sample size	CS	US	Manipulation	Manipulation timing	Trials during recall	Extinction—recall delay	Outcome measures
Adams et al. (2025)	123	Coloured circles	Shock	Blood PACAP38 measured. PTSD measured	60 min prior to each phase.	4	7 days	SCR
Antov and Stockhorst (2014)	72	Shapes	Loud noise	Stress induction versus control, menstrual cycle phase. Estradiol and progesterone plasma measured	45 min prior to acquisition, mid-cycle and early follicular. Blood drawn prior to conditioning	12	24 h	SCR^
Asthana et al. (2016)	91	Blue and yellow squares	Scream	Reactivation versus standard extinction, val66met genotyping	Reactivation prior to extinction	16	24 h	SCR^
Bierwirth et al. (2021)	60	Neutral faces	Loud noise	Male versus mid-cycle and hormonal contraceptive women. Estradiol and progesterone saliva measured	Saliva collected prior to conditioning	60	24 h	SCR, EEG*
Davignon et al. (2025)	147	Lamp colours	Shock	Estradiol levels based on menstrual phase and oral contraceptive use history	n/a	8	24 h	SCR, fMRI*
Giménez et al. (2020)	30	Lamp colours	Shock	OCD versus healthy control, CBT treatment. fMRI scan for glutamate levels in vmPFC	Treatment took place after recall. fMRI scan during extinction and recall	8	24 h	SCR, fMRI*
Hagedorn et al. (2022)	60	Shapes	Shock	Hydrocortisone versus placebo, generalization. Cortisol levels measured in saliva	Hydrocortisone or placebo 40 min prior to extinction, generalization during both extinction and recall. Saliva collected before and after drug administration	4	24 h	SCR, fMRI
Hartley et al. (2012)	110	Coloured squares	Shock	5-HTTLPR genotype measured	n/a	20	24 h	SCR*
Li and Graham (2016)	60	Spiders	Shock	Serum estradiol and progesterone, spider phobia versus healthy control	Serum drawn after extinction	7	24 h	SCR, valence, expectancy*
Mayo et al. (2020a), Mayo et al. (2020b)	75	Lamp colours	Loud noise	Stress versus control. AEA, 2-AG, OEA, PEA measured in plasma	Stress or control after extinction. Plasma drawn after during and after stress	8	24 h	FPS^
Mayo et al. (2020a); Mayo et al. (2020b)	45	Lamp colours	Loud noise	FAAH inhibition versus placebo, cortisol, AEA, 2-AG, OEA, PEA measured in plasma	FAAH inhibition or placebo for 8 days prior to conditioning. Plasma drawn after extinction and recall	5	24 h	FPS, SCR, heart rate, valence, arousal^
Milad et al. (2006)	42	Lamp colours	Shock	Early versus late follicular women versus men	n/a	10	24 h	SCR*
Milad et al. (2010)	36	Lamp colours	Shock	Serum estradiol and progesterone	Blood drawn prior to acquisition and recall	5	24 h	SCR*
Milligan-Saville and Graham (2016)	64	Neutral faces	Shock	Mothers versus non-mothers. Serum estradiol measured	Blood drawn 15 min after extinction	7	24 h	SCR*
Merz et al. (2018)	40	Lamp colours	Shock	Hydrocortisone versus placebo. Saliva cortisol measured	Hydrocortisone or placebo prior to extinction. Saliva before and after drug administration	8	24 h	SCR, fMRI
Ney et al. (2021a); Ney et al. (2021b); Ney et al. (2021c)	191	Coloured circles	Shock	BDNF plasma and Val66Met measured	Plasma collected after extinction	10	2 days	SCR
Panitz et al. (2018)	87	Neutral faces	Loud noise	Val158Met measured	n/a	60	24 h	SCR, EEG, heart rate, valence, arousal
Peyrot et al. (2024)	168	Lamp colours	Shock	Oral contraceptive use versus follicular versus luteal phase versus male. Stress induction	Stress induction prior to extinction	8	24 h	SCR
Shi et al. (2018b), Shi et al. (2018a), Shi et al. (2018c)	43	Red and yellow squares	Shock	Extinction conducted either 24 h or 2 weeks after acquisition. Plasma orexin A measured	Extinction conducted either 24 h or 2 weeks after acquisition. Blood taken 30 min before extinction	10	24 h	SCR^
Shi et al. (2018b); Shi et al. (2018a); Shi et al. (2018c)	91	Red and yellow squares	Shock	ADRB2 genotype measured	n/a	20	24 h	SCR^
Spohrs et al. (2022)	55	Shapes	Thermal	FAAH C385A measured	n/a	20	24 h	fMRI, fear ratings
White and Graham (2016)	73	Neutral faces	Shock	Serum estradiol and progesterone	Blood drawn prior to acquisition	7	24 h	SCR, valence, expectancy, fear ratings*
Zabik et al. (2021)	59	Coloured bag images	Snake (virtual reality)	FAAH C385A measured	n/a	20	24 h	SCR, fMRI, expectancy
Zeidan et al. (2011)	34	Coloured lamps	Shock	Naturally cycling women, serum estradiol and progesterone measured	Blood drawn prior to extinction	8	24 h	SCR, fMRI*

*Use of extinction retention index, ^use of correction to extinction learning. 2-AG = 2-arachidonoyl glycerol, AEA, arachidonoyl ethanolamide; BDNF, brain-derived neurotropic factor; CBT, cognitive behavioural therapy; EEG, electroencephalography; fMRI, functional magnetic resonance imaging; FPS, fear potentiated startle; FAAH, fatty acid amide hydrolase; h, hours; OCD, obsessive compulsive disorder; OEA, oleoylethanolamide; PACAP, Pituitary adenylate cyclase activating polypeptide; PEA, palmitoylethanolamide; PTSD, posttraumatic stress disorder; SCR, skin conductance response; vmPFC, ventromedial prefrontal cortex.

Other studies have measured peripheral levels and genotyping of different neurotransmitter systems and tested the relationship between these measurements and subsequent extinction retention. Asthana et al. (2016) found that the risk allele of the Brain-derived neurotrophic factor (BDNF) Val66Met polymorphism was associated with deleterious effects on extinction retention following memory reconsolidation. Poorer retention of extinction memory in Met carriers has been reported to be associated with low but not high BDNF plasma levels (Ney et al., 2021b). Similar associations have been found between impaired extinction retention and risk alleles of the serotonin transporter signalling polyadenylation (Hartley et al., 2012), fatty acid amide hydrolase (Mayo et al., 2020a; Spohrs et al., 2022; Zabik et al., 2021), and β2-adrenergic receptor, *η_p_^2^* = 0.09 (Shi et al., 2018a), whereas catechol-O-methyltransferase had effects on fear bradycardia (*d* = 0.62) but not SCR (Panitz et al., 2018). AC heterozygotes of the FAAH rs324420 single-nucleotide polymorphism also had increased dACC activity during extinction retention in a separate study (Spohrs et al., 2022). Two studies have also tested the relationship between glutamate and orexin blood levels and extinction retention. These studies reported significantly impaired extinction retention in participants with higher glutamate (Giménez et al., 2020) and lower orexin (Shi et al., 2018b) levels, though the latter effect was only significant in 24-h but not two-week extinction retention tests. No relationship was found between blood pituitary adenylate cyclase activating polypeptide (PACAP) levels and extinction retention, except for in a small group of female participants with high PACAP, high PTSD symptomology, and impaired extinction retention (Adams et al., 2025).

Overall, these studies portray a complex but emerging picture of the effect of several neurotransmitter systems on fear extinction retention. The strongest available evidence in the neurotransmitter field suggests that estradiol plays a role in the retention of extinction memories. Future research is needed to discern under what conditions these systems may be targeted to effectively improve treatments for anxiety and traumatic disorders.

**Table:** 

Summary—neurotransmitters
Limited studies have investigated the relationship between neurotransmitters and spontaneous recovery
Available research suggests that estradiol, BDNF, serotonin, orexin, the β2-adrenergic receptor, endocannabinoids, and glutamate may be associated with extinction retention

### Time delay between extinction and spontaneous recovery

Of the ten studies that controlled for time (Table 8), Dural et al. (2022) found that time between extinction and test had no impact on spontaneous recovery in SCR. However, showing the unreinforced CS+ 10 min prior to extinction promoted extinction retention compared to the six-hour and no reminder groups (*ƞ^2^* = 0.04). This difference was preserved over 1, 15, and 90 day retention gaps (Dural et al., 2022). This manipulation was replicated with a 48-h (Fernandez-Rey et al., 2018) and two week (Klinke et al., 2025) extinction to test window. Similarly, partial reinforcement (occasional CS-US pairings) during extinction led to sustained extinction retention over a one-week period (Culver et al., 2018). Spontaneous recovery was also tested across 24-h and 1-year time gaps by Mueller and Pizzagalli (2016). However, the study was underpowered (*N* = 16) and no CS discrimination (measured by SCR) was observed at either 24-h or 1-year time intervals (Mueller and Pizzagalli, 2016). Szeska et al. (2020) tested spontaneous recovery 1 day and 4 weeks after extinction, and although spontaneous recovery was observed at both time points, it was weaker after 4 weeks compared to 1 day. Spontaneous recovery was also weaker 2 weeks after extinction compared to 1 day (Gong et al., 2025) and non-differential SCRs were lower 1 week compared to 1 day in Kampa et al. (2023), though Bauer et al. (2025) found that participants with PTSD (compared to healthy controls) had significantly different retention between 1 day and 1 month retention intervals.

**Table 8:** Use of spontaneous recovery paradigms with varying time intervals between extinction and spontaneous recovery test.

Study	Sample size	CS	US	Manipulation	Manipulation timing	Trials during recall	Extinction—recall delay	Outcome measures
An and Chen (2016)	30	Yellow and blue squares	Loud noise	Time of recall (within-subjects)	n/a	10	1- and 30-day recall tests	SCR
Culver et al. (2018)	39	Neutral faces	Scream	Partial reinforcement extinction versus control	¼ of trials reinforced during extinction	4	1 week	SCR, expectancy, valence^
Deng et al. (2021)	70	Coloured squares	Shock	iTBS to vertex versus iTBS to left dlPFC	Before and after extinction	15	24 h, 1 month	SCR^
Dunning and Hajcak (2015)	115	Rectangles	Shock	Fear generalization	After acquisition and before extinction	10	1 week	FPS, expectancy
Dural et al. (2022)	219	Snakes and spiders, coloured circles	Shock	Time of recall (within-subjects, type of CS, reconsolidation reminder)	Reconsolidation reminder: 10 min or 6 h prior to extinction, or no reminder	11	24 h, 15 days, and 3 months	SCR, arousal^
Fernandez-Rey et al. (2018)	58	Coloured squares	Loud noise	Fear (reactivation versus control)	Reactivation prior to extinction	11	2 days	SCR^
Glenn et al. (2021)	36	Blue and yellow bells, neutral faces	Scream or alarm	Social versus non-social stimuli, youth versus adults, generalization	Generalization during recall	24	3 weeks	SCR, fMRI, threat appraisal, explicit memory
Gold et al. (2020)	200	Neutral faces	Scream	Anxiety versus healthy control, generalization	Generalization during recall	24	3 weeks	SCR, fMRI, threat appraisal, explicit memory
Gong et al. (2025)	96	Coloured squares	Shock	Social support intervention versus control	Before extinction	15	24 h, 2 weeks	SCR, fMRI^
Haaker et al. (2013a), Haaker et al. (2013b)	85	Shapes	Shock	n/a	n/a	6	6 days	SCR, FPS, fear ratings^
Haaker et al. (2015)	41	Shapes	Shock	L-DOPA versus placebo	L-DOPA or placebo immediately following extinction	6	1 week	SCR, fMRI, fear ratings
Kampa et al. (2023)	103	Coloured squares	Shock	Immediate versus delayed extinction	Delayed extinction was 24 h delay	16	24 h, 1 week	SCR, fMRI, arousal^
Klein et al. (2021)	65	Neutral faces	Scream	Attention manipulation, generalization	Attention manipulation during extinction, generalization during recall	12	3 days	SCR, FPS, fear ratings, expectancy
Klinke et al. (2025)	113	Coloured shapes	Shock	Stress versus no stress induction	Stress induction 24 h prior to acquisition	16	24 h, 15 days	SCR, FPS, expectancy, fear ratings^
Klumpers et al. (2012)	54	Coloured neutral faces	Shock	DCS, THC versus placebo	DCS, THC, or placebo 2 h prior to extinction	8	2 days	SCR, FPS
Krause-Utz et al. (2016)	43	Blue square, yellow triangle	Shock	Borderline versus healthy control	n/a	18	3 days	SCR, fMRI, valence, arousal
Lonsdorf et al. (2014)	39	Shapes	Shock	Threat context	Before and after reinstatement	6	6 days	SCR, fMRI, fear ratings
Maples-Keller et al. (2022a)	34	Coloured shapes	Air blast	MDMA versus placebo	Prior to extinction	16	2 days	FPS^
Menz et al. (2016)	80	Shapes	Shock	Sleep manipulated—no sleep versus REM sleep versus SWS sleep. PSG measured.	After extinction learning, split night study.	10	2 days	SCR, fMRI^
Mueller and Pizzagalli (2016)	16	Neutral faces	Loud noise	Time of recall (within-subjects)	n/a	60	24 h, 1 year	SCR, ERP, valence, arousal
Ney et al. (2021a), Ney et al. (2021b), Ney et al. (2021c)	191	Coloured circles	Shock	n/a	n/a	10	2 days	SCR
Ney et al. (2023a), Ney et al. (2023b), Ney et al. (2023c)	110, 109	Words	Loud noise	Level of processing of words: high, low versus none	During extinction	8, 16	48 h	Reaction time, valence, expectancy
Norrholm et al. (2008)	92	Coloured light	Air blast	Delayed (72 h versus immediate extinction, single cue versus differential conditioning)	n/a	4	24 h, 4 days	FPS, expectancy^
Schenker et al. (2022)	248	Coloured circles	Shock	PTSD versus trauma and non-trauma control	n/a	10	2 days	SCR^
Staples-Bradley et al. (2018)	29	Neutral faces	Scream	Multiple contexts presented during extinction	During recall the context from the last third of extinction was presented	4	7 days	Expectancy, fear ratings
Sun (2019)	190	Coloured lamps	Shock	Multiple studies	n/a	2, 8	24 h, 2 days, 7 days	SCR, fMRI
Szeska et al. (2020)	80	Single cue—coloured pentagon	Shock	tVNS versus sham stimulation, reinstatement test	Reinstatement after first recall test. tVNS/sham during extinction	16	20–28 h and then 28 days	FPS, expectancy
Toumbelekis et al. (2018)	61	Coloured squares	Shock	Thinking of positive attachment figure versus positive experience	Prior to conditioning	16	2 days	FPS, expectancy*
van Dis et al. (2019)	87	Neutral faces	Shock, funny film clips	Counter- conditioning versus extinction versus non-contingent positive film clips	After acquisition	2	7 days	SCR, FPS, valence, arousal, fear ratings^
Wicking et al. (2016)	54	Shapes	Shock	PTSD versus trauma and non-trauma controls	Context change to recall	10	7 days	SCR, expectancy
Zbozinek (2017)	58	Neutral faces	Shock	Negative or positive mood induction	Prior to extinction and in between extinction phases	2	7 days	SCR, FPS, expectancy, fear ratings*

*Use of extinction retention index, ^use of correction to extinction learning. CS, conditioned stimulus; DCS, D-cycloserine; dlPFC, dorsolateral prefrontal cortex; fMRI, functional magnetic resonance imaging; FPS, fear potentiated startle; FAAH, fatty acid amide hydrolase; h, hours; iTBS, intermittent theta-burst stimuluation; L-DOPA, levodopa; MDMA, 3,4-Methylenedioxymethamphetamine; PSG, polysomnography; PTSD, posttraumatic stress disorder; REM, rapid eye movement; SCR, skin conductance response; SWS, slow wave sleep; THC, delta9-tetrahydrocannabinol; tVNS, Transcutaneous Vagus Nerve Stimulation.

Norrholm et al. (2008) altered the timing of extinction so that it occurred either immediately (10 min) or delayed (72 h) after acquisition. The extinction retention test for the immediate extinction group was 96 h after extinction whereas the delayed group extinction test was 24 h after extinction. FPS increased significantly for both groups, with the delayed group showing differential fear recall, while the immediate group experienced generalised fear recall (enhanced startle during both CS+ and CS−). The difference in spontaneous recovery may have been due to the discrepant timing of the extinction test relative to extinction rather than that of extinction relative to acquisition (Norrholm et al., 2008).

Counterconditioning had no effect on one-week and same-day spontaneous recovery as measured by US expectancy, SCR, or FPS, though there was substantially higher return of fear during spontaneous recovery measured one-week after extinction compared to same day extinction tests across all outcome measures (van Dis et al., 2019). Cognitive reappraisal techniques have provided mixed outcomes for extinction retention longevity. An and Chen (2016) found strong 30-day extinction retention when reappraisal instructions were applied during extinction (*η^2^* = 0.10) and, to a lesser extent, during acquisition. Sun (2019), however, reported mixed results whereby cognitive reappraisal had no impact on extinction retention when a seven-day gap was present between extinction and extinction test, though with a one-day gap between extinction and test the control group exhibited fear recall in SCRs while fear recall was present in US expectancy across all groups.

Comparing the influence of emotional connection, Toumbelekis et al. (2018) had participants reflect on either positive attachment figures or non-attachment related positive experiences prior to commencing the conditioning paradigm. Measuring spontaneous recovery 48 h later for all participants, the data revealed no fear recall in FPS or US expectancy for both groups (Toumbelekis et al., 2018). 48 h extinction test protocols have also been used by Ney et al. (2021b) and Schenker et al. (2022), though both studies were based on the same dataset and did not find evidence of significant differential fear recall during the extinction test. Klein et al. (2021) used a three-day gap between extinction and extinction test and found that decreased US expectancy and FPS upon spontaneous recovery was associated with increased attention to the CSs during extinction. A six-day gap was used by Lonsdorf et al. (2014) and Haaker et al. (2013b), though while fear ratings indicated fear recall for both cues and the context associated with the US (*η^2^* = 0.18), SCRs showed no fear recall in response to context or cues (*η^2^* = 0.05). Zbozinek (2017) likewise found that no 1-week spontaneous recovery was observed in SCR, self-reported fear ratings, or fear potentiated startle. Finally, Glenn et al. (2021) found that socially relevant USs resulted in significant spontaneous recovery after 3 weeks, CSs conditioned to non-socially relevant USs did not.

In summary, physiological spontaneous recovery might be affected by time, with the weight of evidence suggesting weaker spontaneous recovery if tested more than 24 h after extinction learning, though this effect may be strengthened with more relevant stimuli—such as socially relevant stimuli (for example, faces), and is not replicated across all studies. Future studies are needed to test the hypothesis that a one-day delay between extinction and spontaneous recovery test is the optimal duration for reliable measurement of this type of return of fear.

**Table:** 

Summary—time period between extinction and recall
Not many studies have tested the effect of time between extinction and retention test on spontaneous recovery.
Not all studies suggest so, but on weight, available evidence suggests that 24-h test following extinction may tentatively provide the strongest spontaneous recovery

### General section

The final section of this review will cover other work identified in our search that could not be grouped into substantial categories (Table 9). Firstly, Milad et al. (2005a) is the seminal article in human differential spontaneous recovery research, and the mixed recall and contextual renewal design used in this study has been the foundation for much of the ensuing literature in this field. However, spontaneous recovery across intervals up to 3 h using a non-differential conditioning paradigm was first explored in the 1930’s (Ellson, 1939). Zeidan et al. (2012) assessed the test–retest reliability of the more modern paradigm and found that, when repeated by participants three times at 12-week intervals, spontaneous recovery was correlated within participants and was not significantly different over time. Other outcome measures for these paradigms outside of the standard measures (fMRI, SCR, FPS, subjective ratings) have been tested. Cortical event-related potentials and slow pupil dilations have been shown to index spontaneous recovery (Antov et al., 2020; Bierwirth et al., 2023; Leuchs et al., 2019; Mueller et al., 2014; Mueller and Pizzagalli, 2016), though startle-induced pupil responses (Leuchs et al., 2019) and electrocardiogram during virtual reality (Gromer et al., 2025) did not.

**Table 9:** Use of spontaneous recovery paradigms with other manipulations unclassified above.

Study	Sample size	CS	US	Manipulation	Manipulation timing	Trials during recall	Extinction—recall delay	Outcome measures
Antov et al. (2020)	19	Gabor patches	Loud noise	Generalization	Generalization occurred in each phase	16	24 h	SCR, EEG, valence, arousal, expectancy
Battaglia et al. (2018)	48	Plant, lamp	Shock	Participants were either younger or older adults	n/a	10	24 h	SCR, fear ratings
Bierwirth et al. (2023)	40	Neutral faces	Loud noise	Immediate versus delayed extinction	Extinction either 10 min or 24 h following acquisition	60	24 h	SCR, EEG, heart rate, valence, arousal
Forcadell et al. (2017a)	50	Coloured lamps	Shock	Participants had moderate to strong spider fear. Attentional control tested	Attentional control tested prior to conditioning	6	24 h	SCR, FPS, expectancy*
Forcadell et al. (2017b)	50	Coloured lamps	Shock	Participants had moderate to strong spider fear. Treated with single session exposure thesrapy	Exposure therapy completed after recall	6	24 h	SCR, FPS, expectancy*
Gromer et al. (2025)	100	Grey sphere, closeness to screen determined US likelihood	Shock	Virtual reality.	n/a	1	24 h	SCR, ECG, expectancy, fear
Leuchs et al. (2019)	47	Shapes	Shock, air blast	Comparison between outcome measures	n/a	8	24 h	SCR, FPS, pupil size
Martínez et al. (2012)	46	Coloured lamps	Shock	Correlation between recall and psychological and personality questionnaires	Questionnaires completed prior to conditioning	5	24 h	SCR*
Merz et al. (2020)	51	Neutral faces	Shock	Stress versus control. In-group compared to out-group CS faces used	Stress or control after extinction	16	20 min	SCR^
Milad et al. (2005a); Milad et al. (2005b)	30	Coloured lamps	Shock	Order of recall and renewal phases were counterbalanced^#^	n/a	5	24 h	SCR*
Mueller et al. (2014)	42	Neutral faces	Loud noise	EEG measured relative to anterior midcingulate cortex and vmPFC theta and gamma activity	n/a	60	24 h	SCR, EEG, valence, arousal
Mueller and Pizzagalli (2016)	16	Neutral faces	Loud noise	Time of recall (within-subjects)	n/a	60	24 h, 1 year	SCR, EEG, valence, arousal
Ney et al. (2023a), Ney et al. (2023b), Ney et al. (2023c)	110, 109	Words	Loud noise	Level of processing of words: high, low versus none	During extinction	8, 16	48 h	Reaction time, valence, expectancy
Norrholm et al. (2011)	43	Tones or shapes	Air blast	Auditory versus visual CSs	n/a	4	24 h	FPS^
Rosenbaum et al. (2015)	222	Coloured lamps	Shock	OCD, PTSD, schizophrenia versus control. Demographics assessed	n/a	8	24 h	SCR
Sperl et al. (2016)	32	Neutral faces	Loud noise or shock	Comparison between outcome measures and US type	US type varied during acquisition	240	24 h	SCR, heart rate, arousal, valence
Zeidan et al. (2012)	18	Coloured lamps	Shock	Fear conditioning (including recall) completed in full three times	Each re-test separated by at least 12 weeks	5	24 h	SCR*

^#^Many studies, especially using coloured lamps as conditioned stimuli, have adopted the approach of counterbalancing recall with (delayed) contextual renewal phases. *Use of extinction retention index, ^use of correction to extinction learning. CS, conditioned stimuli; ECG, electrocardiogram; EEG, electroencephalography; fMRI, functional magnetic resonance imaging; FPS, fear potentiated startle; h, hours; OCD, obsessive compulsive disorder; PTSD, posttraumatic stress disorder; SCR, skin conductance response; US, unconditioned stimulus; vmPFC, ventromedial prefrontal cortex.

USs with different characteristics affect the strength and quality of fear conditioning (Ney et al., 2023c; Ney et al., 2022d) and this has been found to hold true for spontaneous recovery: Sperl et al. (2016) reported that fear conditioned with a white-noise burst US was more resistant to extinction compared to fear conditioned with an electric shock for arousal ratings (*η_p_^2^* = 0.089) and SCR (*d* = 0.127), but not valence ratings. CSs with differently perceived valence can also affect subsequent fear conditioning and return of fear (Mallan et al., 2013; Ney et al., 2022b; Ney et al., 2022c), though Merz et al. (2020) found that spontaneous recovery of fear was higher for in-group CS faces (compared to out-group CS faces), but this effect may have been driven by incomplete extinction of fear of out-group CS faces. Whether CSs are visual or auditory does not affect spontaneous recovery (Norrholm et al., 2011).

Battaglia et al. (2018) explored whether age affects spontaneous recovery. The results from this study suggested that older participants (60–70) had significantly higher spontaneous recovery compared to younger participants (20–30), as measured by SCRs (*η^2^* = 0.087); however, this finding was not replicated in an online study where probe reaction time, US expectancy, and CS valence were measured (Ney et al., 2023b) or in a laboratory study using SCRs (Rosenbaum et al., 2015). No effects of attentional control (Forcadell et al., 2017a), anxiety, personality traits, and cognitive interference (Martínez et al., 2012) have been found on spontaneous recovery.

**Table:** 

Summary—general
Cortical event-related potentials and slow pupil dilations have been shown to index spontaneous recovery
US type used during the experiment affects spontaneous recovery
Mixed evidence that CS type affects spontaneous recovery

### Overall summary

We conducted a systematic review of the literature on spontaneous recovery of human fear conditioning. Some differences in spontaneous recovery are observed between clinical and healthy populations, with the most robust differences evident between PTSD and healthy participants. Our review of the literature identified that recovery paradigms with a one-day delay between extinction and spontaneous recovery are the most commonly used and there is some evidence that one-day spontaneous recovery paradigms produce the strongest return of fear, though findings are mixed. Potential correlations between performance in spontaneous recovery experiments and real-world efficacy exist in prospective pharmacological interventions. Interestingly, although numerous types of non-pharmacological interventions have been tested using spontaneous recovery, to our knowledge there has been slower uptake of these interventions in clinical trials. Our review also identifies some consistencies in the neurotransmitter and sleep literatures, which may provide insights into which biological systems to target during exposure therapy and what conditions need to be met to enhance exposure therapy outcomes. These factors include topics such as timing of exposure therapy administration, the amount of sleep required for successful exposure, and even the menstrual cycle phase during which exposure should be conducted. However, and as discussed below, there are notable weaknesses in the operationalisation of the paradigm that likely impair progress in the field. Finally, our quality appraisal (Supplementary materials) indicates that the quality of the articles was overall adequate, though there was enough detail lacking from articles that the quality could be considered mixed. This was especially the case for the identification and treatment of confounding variables, which was rarely addressed across the articles. However, it should be noted that there were no adequate critical appraisal tools that covered important aspects of interstudy robustness, such as variability of statistical decision making.

#### Extinction retention index and other issues affecting spontaneous recovery

A controversial issue in the spontaneous recovery paradigm that has emerged over the last several years is the use of the extinction retention index (Lonsdorf et al., 2019; Ney et al., 2018). The extinction retention index (ERI) corrects responses during the spontaneous recovery phase against responses during the acquisition phase to control for individual differences in physiological responsiveness, and converts the ratio to a percentage score of “fear recovery” or inversely “extinction retention” (Milad et al., 2005a). While these corrections make sense in principle and are even arguably important to use given the extent of individual differences in conditioned responding and extinction learning, their application in the literature has been highly inconsistent. Lonsdorf et al. (2019) conducted a systematic review, finding that across only 37 studies, 16 different operationalizations of the ERI had been used. These operationalizations involved changes to the number and nature of trials (acquisition vs. extinction) included in the calculation and it was found that, when applied to the same dataset, there were weak correlations between many of these ERI scores (Lonsdorf et al., 2019). A potential solution is the use of multiverse analyses, which has been proposed elsewhere (Greaves et al., 2024; Lonsdorf et al., 2022; Sjouwerman et al., 2022).

Our review shows that ERIs are widely used and more often in certain fields than others. For example, the ERI is used widely in the neurotransmitter and biological fields. Similarly, different fields have used different paradigms. In the fMRI and neurotransmitter fields, the fear conditioning paradigm using coloured lamp CSs (Milad et al., 2005a) has been widely adopted. However, different paradigms with different stimuli are used in other fields and these paradigms have not been directly compared. Given that it is known that CS and US types differentially affect fear conditioning, direct comparisons should be made in the future. Finally, many studies have counterbalanced assessments of contextual renewal and spontaneous recovery. Since spontaneous recovery does not require a context change between extinction and recall, counterbalanced designs that may assess renewal prior to spontaneous recovery such as described in Milad et al. (2005a) may lead to distortion of the spontaneous recovery data. More work is needed to understand whether factors such as these affect the reliability of findings across studies.

## Limitations, future directions, and conclusion

This review has several limitations that should be considered. The review was not preregistered, and a meta-analysis was not conducted due to substantial heterogeneity in study designs, populations, and outcome measures. The exclusion of non-English publications may have introduced language bias, and the possibility of publication bias cannot be excluded. Additionally, variability in fear conditioning paradigms, outcome measures, and the inconsistent operationalisation of spontaneous recovery across studies limits comparability and may affect the generalisability of findings.

The findings from this review point to several important future directions for the research field. Firstly, the relationship between performance in spontaneous recovery experiments and real-world clinical outcomes needs to be more thoroughly evaluated. If the validity of these paradigms to represent real-world outcomes can be firmly and robustly established, then many of the existing findings reviewed in this article should provide benefits when translated to therapy. Secondly, some factors in this experimental paradigm deserve more extensive exploration. More studies could examine the effects of the time period between extinction learning and spontaneous recovery to support the hypothesis that one-day delays produce the most reliable return of fear. Likewise, if we are to apply the findings from all fields of spontaneous recovery research to clinical applications, a significantly improved understanding of the timing of interventions during extinction learning is critical (Ney et al., 2023a). It is also important to recognise that the two most used fear measures in human conditioning (SCR and FPS) frequently provide results that are discordant (Norrholm et al., 2023). This outcome-measure inconsistency currently limits interpretability and cross-study comparability, and may have contributed to the variability of findings in the current review. Finally, during our literature synthesis we found that the heterogeneity of the spontaneous recovery literature presented a significant challenge, as studies vary widely in design, populations, outcome measures, and analytical approaches. This variability limits the extent to which findings can be directly integrated or quantitatively compared and may contribute to the reliance on detailed study-level descriptions within reviews. Greater standardisation in experimental paradigms and reporting practices would facilitate more cohesive synthesis and strengthen the cumulative progress of the field (Bach et al., 2023).

In conclusion, this paper reviewed the extant literature on the spontaneous recovery paradigm in fear conditioning. The paradigm has been used in a variety of different ways and for different purposes and appears to have contributed to incremental knowledge gains across a number of fields. However, our review also reveals that the literature is broad, heterogeneous, and promising, but not yet methodologically mature. We were not able to provide quantitative estimates within subsections of the paper due to the extreme heterogeneity across studies. Significant efforts are needed to establish a coherent, transparent, robust, and consistent framework for studying human fear and its spontaneous recovery.

**Table:** 

Summary—critical take away points
The review finds that the paradigm can be used in a variety of different ways to meet the needs of specific research fields
How well the paradigm maps to anxiety disorders is unclear
How well the paradigm prospectively predicts outcomes in exposure therapies has not been adequately explored
Many experimental design factors that may influence outcomes in the paradigm have not been explored. These include things such as CS and US choice, and the timing between extinction and spontaneous recovery.
The importance of timing of intervention relative to extinction learning has not been adequately explored. It is essential to improve our understanding of this issue if we want to achieve translation to clinical practice
The use of flexible data approaches is widespread and an adequate answer to this issue has not been adopted yet
